# Myeloproliferative neoplasm-driving *Calr* frameshift promotes the development of pulmonary hypertension in mice

**DOI:** 10.1186/s13045-021-01064-8

**Published:** 2021-03-30

**Authors:** Keiji Minakawa, Tetsuro Yokokawa, Koki Ueda, Osamu Nakajima, Tomofumi Misaka, Yusuke Kimishima, Kento Wada, Yusuke Tomita, Saori Miura, Yuka Sato, Kosaku Mimura, Koichi Sugimoto, Kazuhiko Nakazato, Kenneth E. Nollet, Kazuei Ogawa, Takayuki Ikezoe, Yuko Hashimoto, Yasuchika Takeishi, Kazuhiko Ikeda

**Affiliations:** 1 Department of Blood Transfusion and Transplantation Immunology, School of Medicine, Fukushima Medical University, 1 Hikarigaoka, Fukushima, 960-1295 Japan; 2 Department of Cardiovascular Medicine, School of Medicine, Fukushima Medical University, Fukushima, Japan; 3 Center for Molecular Genetics, Yamagata University, Yamagata, Japan; 4 Department of Pulmonary Hypertension, School of Medicine, Fukushima Medical University, Fukushima, Japan; 5 Department of Hematology, School of Medicine, Fukushima Medical University, Fukushima, Japan; 6 Department of Diagnostic Pathology, School of Medicine, Fukushima Medical University, Fukushima, Japan

**Keywords:** CALR, Pulmonary hypertension, Myeloproliferative neoplasms, Macrophage, Essential thrombocythemia

## Abstract

Frameshifts in the *Calreticulin* (*CALR*) exon 9 provide a recurrent driver mutation of essential thrombocythemia (ET) and primary myelofibrosis among myeloproliferative neoplasms (MPNs). Here, we generated knock-in mice with murine *Calr* exon 9 mimicking the human *CALR* mutations, using the CRISPR-Cas9 method. Knock-in mice with del10 [*Calr*^del10/WT (wild−type)^ mice] exhibited an ET phenotype with increases of peripheral blood (PB) platelets and leukocytes, and accumulation of megakaryocytes in bone marrow (BM), while those with ins2 (*Calr*^ins2/WT^ mice) showed a slight splenic enlargement. Phosphorylated STAT3 (pSTAT3) was upregulated in BM cells of both knock-in mice. In BM transplantation (BMT) recipients from *Calr*^del10/WT^ mice, although PB cell counts were not different from those in BMT recipients from *Calr*^WT/WT^ mice, *Calr*^del10/WT^ BM-derived macrophages exhibited elevations of pSTAT3 and Endothelin-1 levels. Strikingly, BMT recipients from *Calr*^del10/WT^ mice developed more severe pulmonary hypertension (PH)—which often arises as a comorbidity in patients with MPNs—than BMT recipients from *Calr*^WT/WT^ mice, with pulmonary arterial remodeling accompanied by an accumulation of donor-derived macrophages in response to chronic hypoxia. In conclusion, our murine model with the frameshifted murine *Calr* presented an ET phenotype analogous to human MPNs in molecular mechanisms and cardiovascular complications such as PH.

**Supplementary Information:**

The online version contains supplementary material available at 10.1186/s13045-021-01064-8.

## To the editor,

**Fig. 1:**
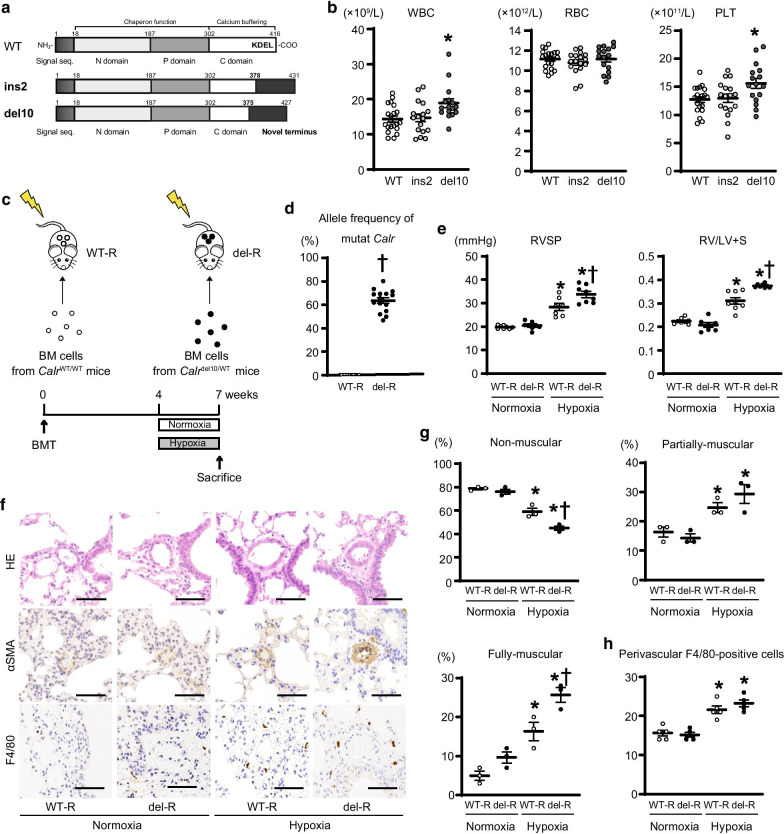
Hematopoietic cells with *Calr* mutation exacerbate the development of pulmonary hypertension in response to chronic hypoxia. **a** The knock-in mice with C57BL/6 J background carrying frameshifted murine *Calr*, del10 (*Calr*^del10/WT^ mice) and ins2 (*Calr*^ins2/WT^ mice) were generated using the CRISPR-Cas9 method. Structure of wild-type (WT) and frameshifted murine CALR proteins are shown. Both generated mutant proteins with shortened calcium-buffering sites and absent KDEL sequence, which is the signal to retain the CALR protein in the endoplasmic reticulum. **b** Leukocyte (white blood cell) counts (WBC), red blood cell counts (RBC), and platelet counts (PLT) in WT mice (*Calr*^WT/WT^ mice, n = 21), *Calr*^ins2/WT^ mice (n = 17), and *Calr*^del10/WT^ mice (n = 16) in the peripheral blood. ^*^P < 0.05 versus the WT group. **c** Schematic diagram of the experimental design of bone marrow (BM) transplantation (BMT). BM cells from control *Calr*^WT/WT^ mice or *Calr*^del10/WT^ mice were injected into the lethally irradiated WT mice (C57BL/6 J mice). Four weeks after BMT, the recipient mice transplanted with the BM cells from the *Calr*^WT/WT^ mice (WT-R) or *Calr*^del10/WT^ mice (del-R) were subjected to normoxia (21% O_2_) or chronic hypoxia (10% O_2_) for 3 weeks. **d** Allele frequency of the mutant *Calr* in the peripheral leukocytes of recipient mice at 4 weeks after BMT (n = 15, each). **e** Right ventricular (RV) systolic pressure (RVSP) and RV hypertrophy determined by dividing the RV weight by the left ventricular weight including the septum (RV/LV + S) (n = 6–8). **f** Representative hematoxylin–eosin (HE) staining and immunohistochemistry with antibodies to anti-α smooth muscle actin (αSMA) and anti-F4/80 images in the lung of BMT recipient mice from *Calr*^WT/WT^ or *Calr*^del10/WT^ mice. Scale bars, 50 µm. **g** Quantitative analysis of the percentage of muscularized distal pulmonary arteries in αSMA-immunostained sections (n = 3, each). **h** Quantitative analysis of the pulmonary perivascular macrophages determined as F4/80-positive cells, per 30 vessels (n = 5, each). Data are presented as means ± SEM. **d, e, g, h** ^*^P < 0.05 versus the corresponding normoxia group and ^†^*P* < 0.05 versus the corresponding BMT recipient mice from *Calr*^WT/WT^ mice. WT-R, recipient mice transplanted with BM cells from *Calr*^WT/WT^ mice; del-R, recipient mice transplanted with BM cells from *Calr*^del10/WT^ mice. Oligonucleotides and antibodies used are listed in Additional files 8, 9

*CALR* frameshifts provide a recurrent myeloproliferative neoplasm (MPN) driver [1]. Pulmonary hypertension (PH) is a life-threatening cardiopulmonary disease characterized by increased pulmonary arterial (PA) pressure. Bone marrow (BM)-derived cells and perivascular inflammatory infiltrates contribute to PA remodeling in PH [2, 3]. Among 5 etiological groups, the WHO group-V PH encompasses multifactorial mechanisms, including MPNs, which are often complicated by PH, with 5%-60% of the prevalence [4–6]. MPN-related PH is associated with crucial features, such as thromboembolism and hypermetabolic state [5]. However, the association of PH with *CALR* mutation remains uncertain. Here, we generated *Calr*^del10/WT^ and *Calr*^ins2/WT^ knock-in mice (Fig. 1a, Additional file 1: Fig. S1), investigated their hematopoiesis, and clarified the role of hematopoietic *Calr* mutation in PH using BM transplantation (BMT) and chronic hypoxia, which provokes PH [7].

In a public database [8], 138 *CALR* frameshifts, including the major del52 and ins5 [1], and another frameshift in 2 MPN patients (ET, myelofibrosis) exactly matching the *Calr*-del10, have been noted in patients with hematopoietic cancers, mostly MPNs (Additional file 7: Table S1, Additional file 1: Fig. S1f). Mouse models carrying frameshifted *CALR* showed ET or, rarely, myelofibrosis [9]. Likewise, *Calr*^del10/WT^ mice developed ET with phosphorylated STAT3 (pSTAT3) and cell-surface thrombopoietin-receptor (TpoR) expressions suggested to accompany mutant CALR [10], whereas *Calr*^ins2/WT^ mice showed a slight splenic enlargement (Fig. 1b, Additional files 2, 3: Fig. S2-S3).

To elucidate the roles of hematopoietic *Calr* mutation in PH, we performed non-competitive BMT from *Calr*^del10/WT^ mice (Fig. 1c), as we reconstituted *Jak2*V617F^+^ MPNs [11]. At 4 weeks after BMT, the engraftments were achieved in the BMT recipients from *Calr*^del10/WT^ mice (del-R, Fig. 1d), but their PB cell counts (Additional files 4: Fig. S4) and BM megakaryocytic distribution did not differ from BMT recipients from *Calr*^WT/WT^ mice (WT-R). We assessed right heart hemodynamics and right ventricular (RV) hypertrophy, showing that neither RV systolic pressure (RVSP) nor right ventricle/left ventricle-plus-septum weight ratio (RV/LV + S) differed between WT-R and del-R. Subsequently, del-R were exposed to chronic hypoxia (10% O_2_) for 3 weeks. Strikingly, although chronic hypoxia elevated RVSP and RV/LV + S in both WT-R and del-R, these levels in del-R were significantly greater than in WT-R, suggesting that hematopoietic *Calr* mutation promotes PH (Fig. 1c-e).

**Fig. 2:**
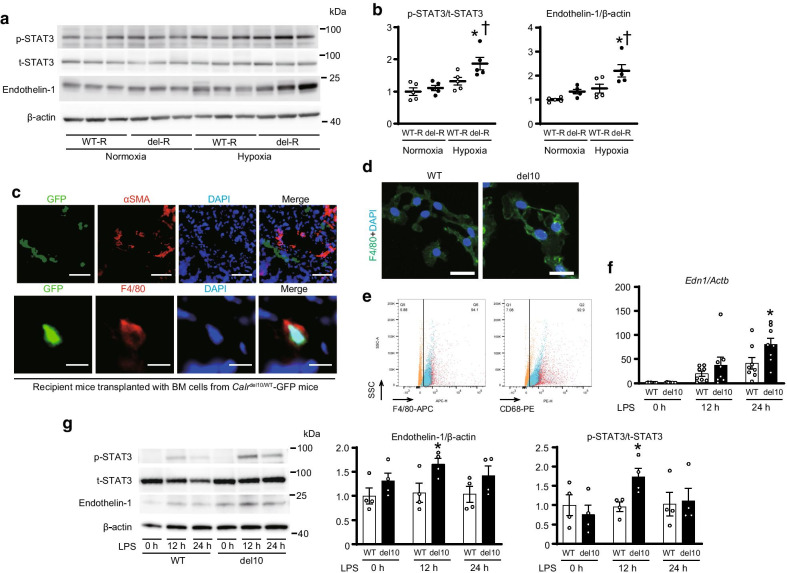
STAT3 phosphorylation and Endothelin-1 expression in the lung and macrophages from *Calr*^del10/WT^ mice. **a** Western blot of lung homogenates of the BMT recipients from *Calr*^WT/WT^ mice (WT-R) or *Calr*^del10/WT^ mice (del-R), immunoblotted with the indicated antibodies. **b** Phosphorylated STAT3 (p-STAT3) to total STAT3 (t-STAT3) or Endothelin-1 to β-actin ratios are shown in the graphs. The average value for WT-R under normoxia was set to 1 (n = 5, each). ^*^P < 0.05 versus the corresponding normoxia group and ^†^*P* < 0.05 versus the corresponding WT-R. **c** The lethally irradiated WT C57BL/6 J mice were transplanted with the BM cells from *Calr*^del10/WT^/CAG-EGFP mice. These recipient mice were subjected to chronic hypoxia for 3 weeks, and then the lungs were fixed and stained with the indicated antibodies. Upper images show representative immunofluorescence of the lung sections stained with anti-GFP (green) and anti-αSMA (red) antibodies and DAPI (blue). Scale bars, 50 µm. Lower images show representative immunofluorescence of the lung sections stained with anti-GFP (green) and anti-F4/80 (red) antibodies and DAPI (blue). Scale bars, 10 µm. **d-g** BM mononuclear cells isolated from the *Calr*^WT/WT^ or *Calr*^del10/WT^ mice were cultured in the presence of 10 ng/mL of M-CSF for 6 days. **d** Representative immunofluorescence images of the cells stained with anti-F4/80 (green) and DAPI (blue) are shown. More than 90% of cells were macrophages expressing F4/80. Scale bars, 25 µm. **e** Dot plot of flow cytometry for cultured macrophages. Red, blue, and orange dots represent cells from *Calr*^WT/WT^ mice, *Calr*^del10/WT^ mice, and negative control (mixture of WT and *Calr* del10 cells), respectively. Over 90% WT and del10 cells were positive for both F4/80 and CD68. SSC indicates side scatter. **f** The cultured macrophages were then stimulated with 0.05 µg/mL of lipopolysaccharide (LPS), a potent activator of macrophages. The mRNA expression levels of *Endothelin-1* (*Edn1*) were analyzed at the indicated time (n = 8, each). *Actb* was used for normalization. The average value for the macrophages from *Calr*^WT/WT^ mice at baseline was set to 1. **g** Left panels show western blots on STAT3, Endothelin-1, and β-actin in the macrophages stimulated with 0.05 µg/mL of LPS. Right graphs show phosphorylated STAT3 (p-STAT3) to total STAT3 (t-STAT3) or Endothelin-1 to β-actin ratios at the indicated time. The average value for the macrophages from *Calr*^WT/WT^ mice at the baseline was set to 1 (n = 4, each). All data are presented as means ± SEM. ^*^P < 0.05 versus the corresponding WT group. WT, macrophages derived from the *Calr*^WT/WT^ mice; del10, macrophages from the *Calr*^del10/WT^ mice. Oligonucleotides and antibodies used are listed in Additional files 8, 9

Lung histology showed significant increases in PA medial wall thickness and muscularization, indicated by α smooth muscle actin, without thrombosis in del-R compared to WT-R under chronic hypoxia, whereas F4/80^+^ macrophages rather than TpoR^+^ cells were increased specifically in PA regions in both WT-R and del-R (Fig. 1f-h, Additional file 3: Fig. S3e). However, pSTAT3 levels were elevated in the lungs of del-R compared to WT-R after chronic hypoxia. The expression of Endothelin-1, an important vasoactive peptide involving PA remodeling in PH [4, 5], was also increased in the lungs of del-R compared to WT-R under chronic hypoxia (Fig. 2a, b, Additional file 5: Fig. S5). We visualized the *Calr*^del10/WT^ BM-derived cells using CAG-EGFP: in the lungs of BMT recipients from *Calr*^del10/WT^/CAG-EGFP mice, donor-derived macrophages accumulated in PA regions, but donor-derived cells were not observed in vascular walls (Fig. 2c), suggesting that *Calr*^del10/WT^ BM-derived macrophages migrated into the PA regions.

RNA sequencing of hematopoietic progenitors showed *Calr*-del10 activated JAK-STAT pathway, as well as cardiac-hypertrophy pathway that includes upregulation of *Endothelin-1*. Also, human *CALR*-del52 introduction upregulated *Endothelin-1* in a macrophage cell line (Additional file 6: Fig. S6). We next obtained *Calr*^del10/WT^ macrophages by culturing BM-mononuclear cells (BM-MNCs) in the presence of M-CSF (Fig. 2d, e). The increases in the *Endothelin-1* and pSTAT3 levels did not show the statistical difference between in *Calr*^WT/WT^ and *Calr*^del10/WT^ macrophages at baseline, but these levels in *Calr*^del10/WT^ macrophages were significantly more upregulated compared to *Calr*^WT/WT^ macrophages after lipopolysaccharide stimulation (Fig. 2f, g). These data suggest that BM-derived macrophages with the *Calr* mutation played important roles in the PA remodeling.

To date, most of studies for PH in MPN patients lack information about driver mutations, although a retrospective study indicated higher prevalence of *CALR* mutations in ET patients with PH than those without [6]. Besides megakaryocyte lineage with TpoR expression, a recent study indicated that transcriptional misregulation occurs with JAK-STAT activation in *CALR*-mutated PB-MNCs similar to *JAK2*-mutated PB-MNCs [12]. Our murine model revealed a hematopoietic phenotype with relevance to human MPNs with *CALR* mutations in terms of molecular mechanisms and PH. Further study of associations between *CALR* mutations and PH or macrophage activation is needed (Additional file 10).

## Supplementary Information

**Additional file 1. Fig. S1:** CALR proteins coded by ins2 and del10 frameshifts in murine *Calr* mimicked a feature of those coded by human type 2-like *CALR* mutations that generated novel C termini. **a** Western blot of BM cells using antibody specific for the CALR N terminus (CALR-N) or C terminus (CALR-C). **b** Isoelectric point (pI) in human and murine CALR proteins. **c** Alignment of C domains in mutant murine CALR from codon A352. Acidic and basic residues are in blue and red, respectively. #: the negatively charged amino acid stretches. ^†^: the subjects of previously reported murine CALR mutants. **d-f** Identity and similarity between murine and human CALR frameshifts. The 2 MPN patients, with a mutated protein as CALR p.K375Rfs*52 (c.1124_1133del), matched the murine *Calr* del10 (p.K375Rfs*52 coded by *Calr* c.1124_1133del), although identity and similarity of the peptides were slightly different (**f**).

**Additional file 2. Fig. S2:** MPN-like phenotypes in knock-in mice with *Calr* frameshifts. **a-b** Body (**a**) and spleen (**b**) weights (*n* = 15—19). **c** BM nuclear cell counts (*n* = 4–6). **d** The proportions of BM CD71^+^Ter119^+^ erythroblasts, Gr1^+^ myeloid cells, B220^+^ B cells, and TCR^+^ T cells in flow cytometry (*n* = 3 in each). **e–f** Histology of BM (**e**) and spleens (**f**). **g-h** The numbers of megakaryocytes per high-power field (HPF) in BM (*n* = 3 in each) and spleens (*n* = 4—10). (*P < 0.05, **P < 0.01).

**Additional file 3. Fig. S3:** Phosphorylation of STAT3 and expression of MPL, thrombopoietin receptor (TpoR). **a** Western blot of whole BM nuclear cells suspended in the absence of exogenous cytokines. **b-c** Flow cytometry gated with a lineage^−^ fraction in BM cells. **b** Overall expression of cell-surface TpoR. Left: Histogram; right: mean fluorescence intensity (MFI). **c** Cell-surface expressions of TpoR and CALR. Left: heatmap plots; right: proportions of cell-surface TpoR^+^ cells in association with CALR expression (*n* = 3 in each experiment; *P < 0.05; ns: no significant difference). **d** Immunofluorescence for MPL in bone marrow. **e** Immunofluorescence for MPL in lung. **d-e** Scale bars, 50 µm.

**Additional file 4. Fig. S4:** Peripheral blood cell counts in the BMT recipients exposed to normoxia or chronic hypoxia for 3 weeks (n = 4–6). (*P < 0.05 versus the corresponding normoxia group).

**Additional file 5. Fig. S5:** Relative *Edn1* mRNA expression levels in the lung (*n* = 5, each). The average value for WT-R mice under normoxia was set to 1. (*P < 0.05 versus the corresponding normoxia group, and ^†^P versus the corresponding WT-R mice under chronic hypoxia)

**Additional file 6. Fig. S6:** Gene expressions. **a-e** RNA sequencing (RNAseq) in LSK (lineage^–^Sca1^+^c-Kit^+^) cells of an aliquot from 4 male mice of 3 months age in each sample from *Calr*^ins2/WT^ mice, *Calr*^del10/WT^ mice, and *Calr*^WT/WT^ mice. **a** Principle component analysis. **b** Venn diagrams of upregulated and downregulated genes (> twofold) in LSK cells of *Calr*^del10/WT^ mice or *Calr*^ins2/WT^ mice relative to those of *Calr*^WT/WT^ mice. **c** Pathway analysis by the Ingenuity Pathway Analysis software (Qiagen). All the pathways in the comparison analysis of canonical pathways with both Z score ≥|2| and *p* < 0.05 in at least one of the *Calr*^ins2/WT^ mice and *Calr*^del10/WT^ mice relative to *Calr*^WT/WT^ mice are shown. • indicates the box which did not reach the level of Z score ≥|2| in the genotype shown. The allow indicates Cardiac Hypertrophy Signaling pathway upregulated in both *Calr*^ins2/WT^ mice and *Calr*^del10/WT^ mice. **d** Individual genes in the Cardiac Hypertrophy Signaling pathway. Differentially expressed genes ( >|10|-fold) in *Calr*^del10/WT^ mice relative to *Calr*^WT/WT^ mice, including *EDN1* that codes Endothelin-1 (allow), are shown. **e** Gene set enrichment analysis (GSEA) for the JAK-STAT pathway. NES indicates normalized enrichment score; FDRq, false discovery rate q value. **f-g** Introduction of FLAG-Tag-inserted human WT and del52 CALR constructs into a macrophage cell line, RAW 264.7. **f** Western blots. **g** The levels of *Endothelin-1* mRNA (*Edn1*) were analyzed in RAW 264.7 cells introduced with *CALR* WT or del52 after incubation under normoxia (21% O_2_) or hypoxia (10% O_2_) for 24 h. Samples were taken from 3 wells for each experiment. *Actb* was used for normalization. The average value for cells introduced with WT CALR and incubated under normoxia was set to 1.

**Additional file 7. Table S1:** Frameshifts in CALR exon 9 on the COSMIC database in hematopoietic cancers.

**Additional file 8. Table S2:** Oligonucleotides used in this study.

**Additional file 9. Table S3:** Antibodies used in this study.

**Additional file 10:** Supplementary methods, results, and references.

## Data Availability

The RNA sequencing data have been deposited in the Gene Expression Omnibus database (GSE152482).

## Abbreviations

CALR: Calreticulin
ET: Essential thrombocythemia
MPN: Myeloproliferative neoplasm
PB: Peripheral blood
BM: Bone marrow
BMT: Bone marrow transplantation
PA: Pulmonary arterial
PH: Pulmonary hypertension
Del-R: BMT recipients from *Calr*^del10/WT^ mice
MNCs: Mononuclear cells
RV: Right ventricular
RVSP: Right ventricular systolic pressure
RV/LV + S: Right ventricle/left ventricle-plus-septum weight ratio
M-CSF: Macrophage colony-stimulating factor
WT: Wild type
WT-R: BMT recipients from *Calr*^WT/WT^ mice
