# Supplementary material for: Myeloproliferative neoplasm-driving Calr frameshift promotes the development of pulmonary hypertension in mice
Source: J Hematol Oncol. 2021 Mar 30;14:52. doi: 10.1186/s13045-021-01064-8 (PMC8011226; doi:10.1186/s13045-021-01064-8)
Supplement: Supplementary file 1 — Additional file 1. Fig. S1: CALR proteins coded by ins2 and del10 frameshifts in murine Calr mimicked a feature of those coded by human type 2-like CALR mutations that generated novel C termini. a Western blot of BM cells using antibody specific for the CALR N terminus (CALR-N) or C terminus (CALR-C). b Isoelectric point (pI) in human and murine CALR proteins. c Alignment of C domains in mutant murine CALR from codon A352. Acidic and basic residues are in blue and red, respectively. #: the negatively charged amino acid stretches. †: the subjects of previously reported murine CALR mutants. d-f Identity and similarity between murine and human CALR frameshifts. The 2 MPN patients, with a mutated protein as CALR p.K375Rfs*52 (c.1124_1133del), matched the murine Calr del10 (p.K375Rfs*52 coded by Calr c.1124_1133del), although identity and similarity of the peptides were slightly different (f). [file 13045_2021_1064_MOESM1_ESM.pdf]

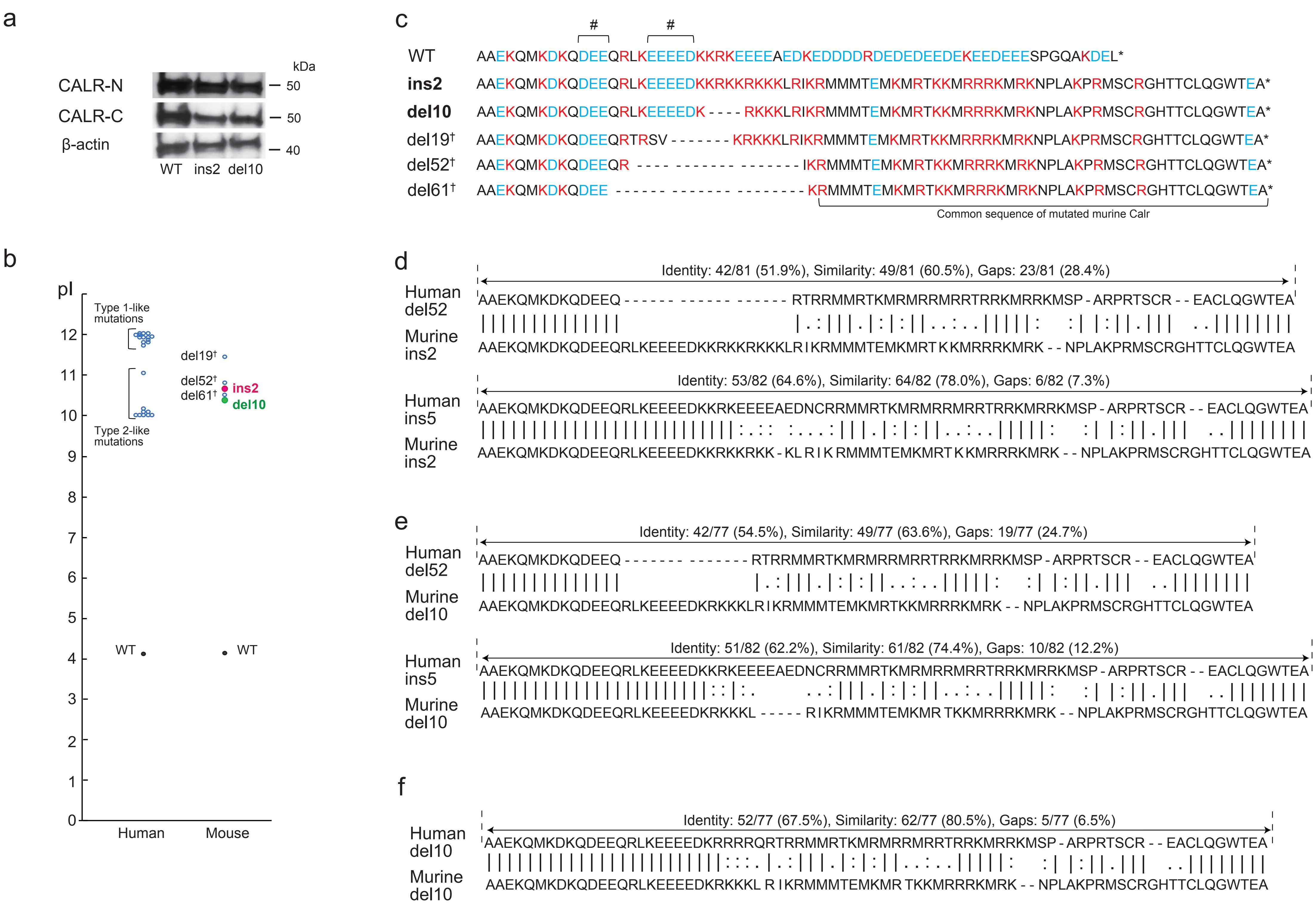

a
kDa
CALR-N
CALR-C
β-actin
50
50
40
WT ins2 del10
b
pI
Type 1-like mutations
Type 2-like mutations
del19†
del52†
del61†
ins2
del10
WT
WT
Human
Mouse
c
#
#
WT AAEKQMKDKQDEEQRLKEEEEDKKRKEEEEAEDKEDDDDRDEDEDEEDEKEEDEEESPGQAKDEL*
ins2 AAEKQMKDKQDEEQRLKEEEEDKKRKKRKKKLRIKRMMMTEMKMRTKKMRRRKMRKNPLAKPRMSCRGHTTCLQGWTEA*
del10 AAEKQMKDKQDEEQRLKEEEEDK - - - - RKKKLRIKRMMMTEMKMRTKKMRRRKMRKNPLAKPRMSCRGHTTCLQGWTEA*
del19† AAEKQMKDKQDEEQRTRSV - - - - - - - - KRKKKLRIKRMMMTEMKMRTKKMRRRKMRKNPLAKPRMSCRGHTTCLQGWTEA*
del52† AAEKQMKDKQDEEQR - - - - IKRMMMTEMKMRTKKMRRRKMRKNPLAKPRMSCRGHTTCLQGWTEA*
del61† AAEKQMKDKQDEE - - - - KRMMMTEMKMRTKKMRRRKMRKNPLAKPRMSCRGHTTCLQGWTEA*
Common sequence of mutated murine Calr
d
Identity: 42/81 (51.9%), Similarity: 49/81 (60.5%), Gaps: 23/81 (28.4%)
Human del52 AAEKQMKDKQDEEQ - - - - RTRRMMRTKMRMRRMRRTRRKMRRKMSP - ARPRTSCR - - EACLQGWTEA
Murine ins2 AAEKQMKDKQDEEQRLKEEEEDKKRKKRKKKLRIKRMMMTEMKMRTKKMRRRKMRK - - NPLAKPRMSCRGHTTCLQGWTEA
Identity: 53/82 (64.6%), Similarity: 64/82 (78.0%), Gaps: 6/82 (7.3%)
Human ins5 AAEKQMKDKQDEEQRLKEEEEDKKRKEEEEAEDNCRRMMRTKMRMRRMRRTRRKMRRKMSP - ARPRTSCR - - EACLQGWTEA
Murine ins2 AAEKQMKDKQDEEQRLKEEEEDKKRKKRKK - KLRIKRMMMTEMKMRTKKMRRRKMRK - - NPLAKPRMSCRGHTTCLQGWTEA
e
Identity: 42/77 (54.5%), Similarity: 49/77 (63.6%), Gaps: 19/77 (24.7%)
Human del52 AAEKQMKDKQDEEQ - - - - RTRRMMRTKMRMRRMRRTRRKMRRKMSP - ARPRTSCR - - EACLQGWTEA
Murine del10 AAEKQMKDKQDEEQRLKEEEEDKRKKKLRIKRMMMTEMKMRTKKMRRRKMRK - - NPLAKPRMSCRGHTTCLQGWTEA
Identity: 51/82 (62.2%), Similarity: 61/82 (74.4%), Gaps: 10/82 (12.2%)
Human ins5 AAEKQMKDKQDEEQRLKEEEEDKKRKEEEEAEDNCRRMMRTKMRMRRMRRTRRKMRRKMSP - ARPRTSCR - - EACLQGWTEA
Murine del10 AAEKQMKDKQDEEQRLKEEEEDKRKKKL - - - - - RIKRMMMTEMKMRTKKMRRRKMRK - - NPLAKPRMSCRGHTTCLQGWTEA
f
Identity: 52/77 (67.5%), Similarity: 62/77 (80.5%), Gaps: 5/77 (6.5%)
Human del10 AAEKQMKDKQDEEQRLKEEEEDKRRRRQRTRRMMRTKMRMRRMRRTRRKMRRKMSP - ARPRTSCR - - EACLQGWTEA
Murine del10 AAEKQMKDKQDEEQRLKEEEEDKRKKKLRIKRMMMTEMKMRTKKMRRRKMRK - - NPLAKPRMSCRGHTTCLQGWTEA
