# Supplementary figures and images for: Myeloproliferative neoplasm-driving Calr frameshift promotes the development of pulmonary hypertension in mice
Source: J Hematol Oncol. 2021 Mar 30;14:52. doi: 10.1186/s13045-021-01064-8 (PMC8011226; doi:10.1186/s13045-021-01064-8)

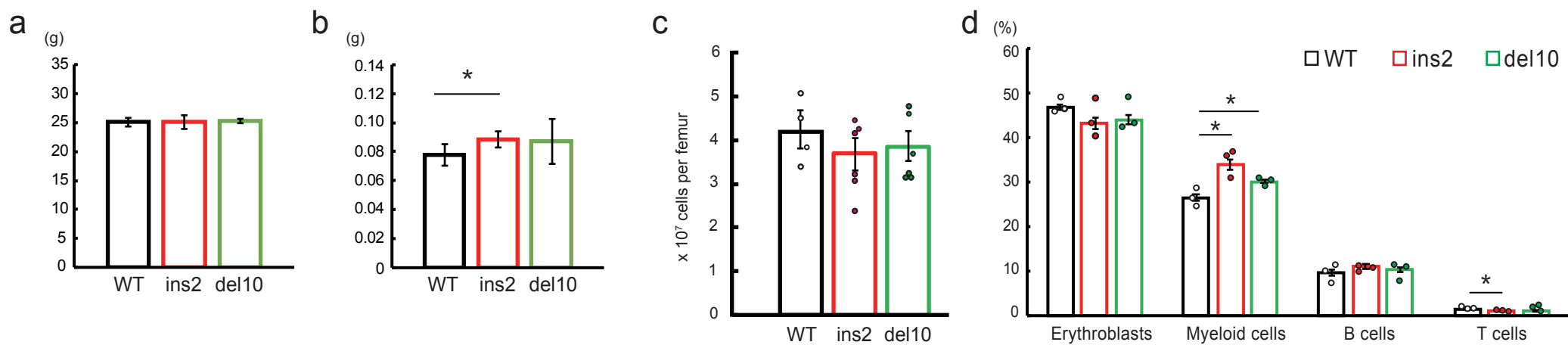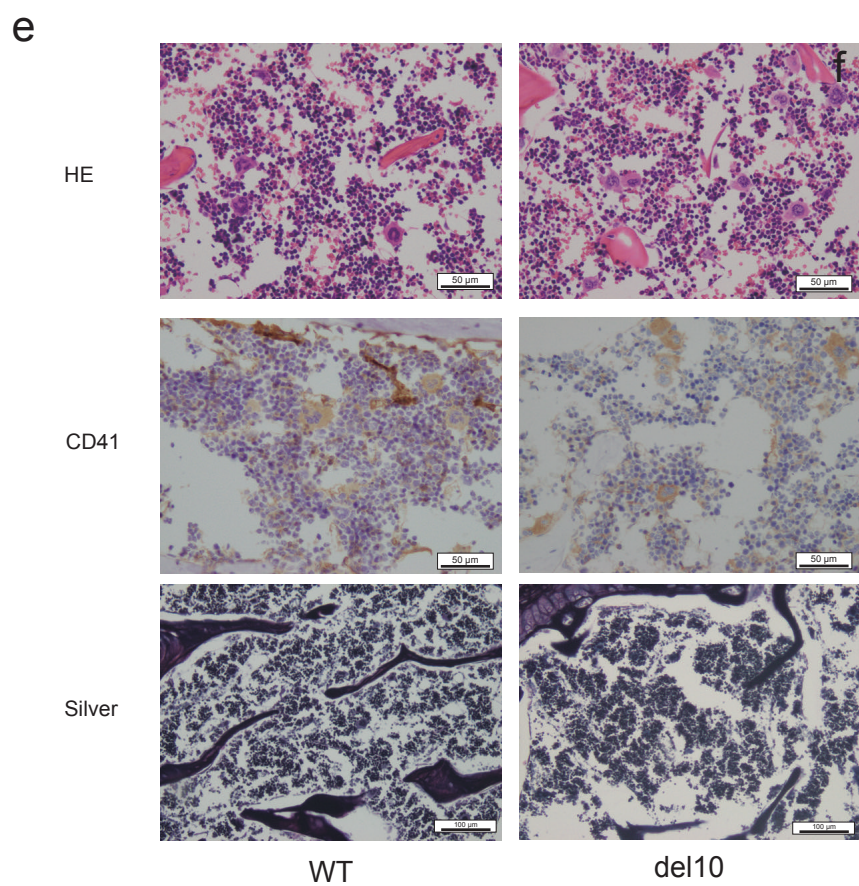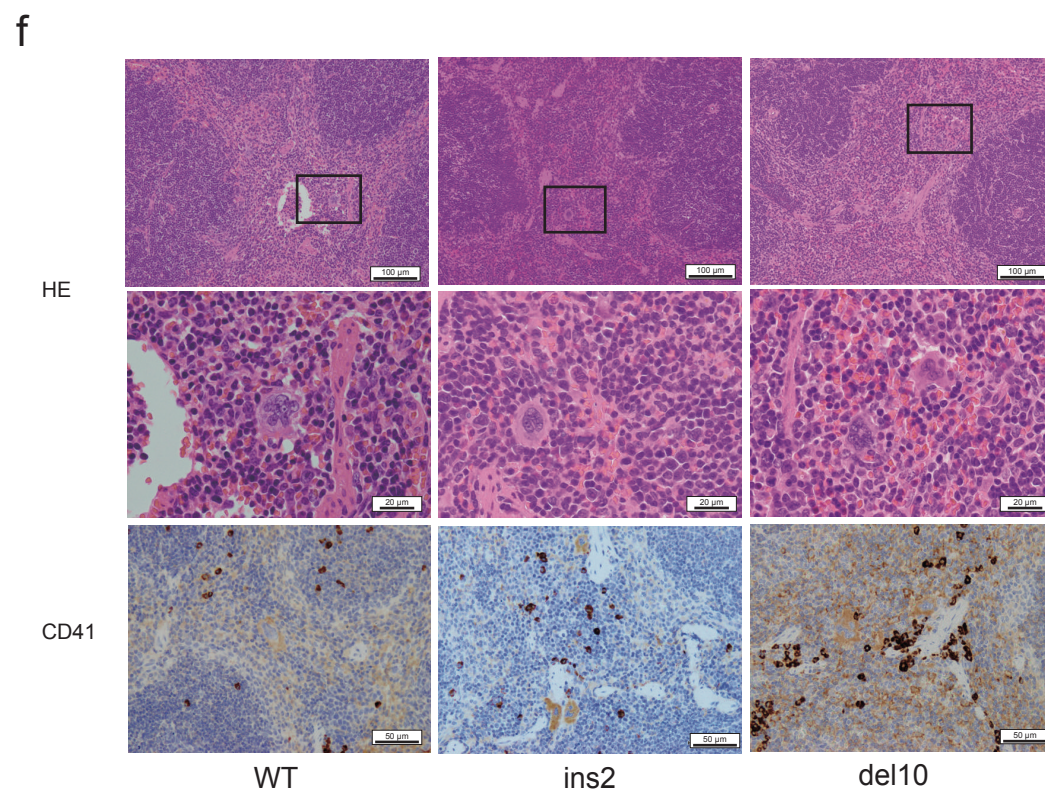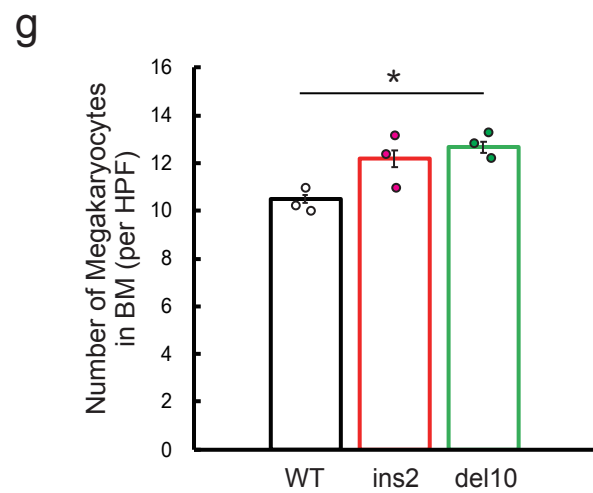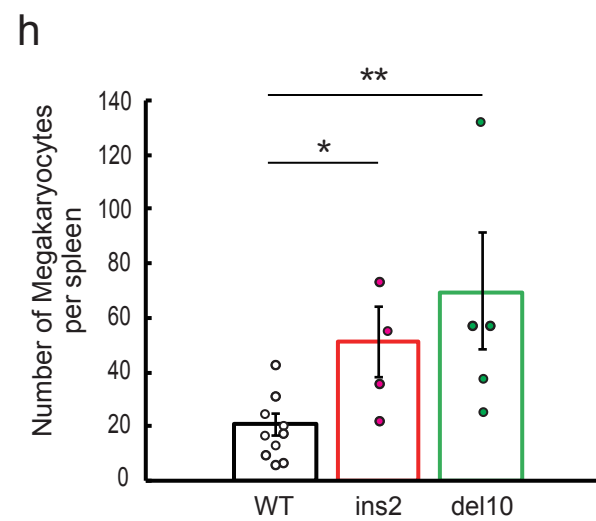

Supplement: Supplementary file 2 — Additional file 2. Fig. S2: MPN-like phenotypes in knock-in mice with Calr frameshifts. a-b Body (a) and spleen (b) weights (n = 15—19). c BM nuclear cell counts (n = 4–6). d The proportions of BM CD71+Ter119+ erythroblasts, Gr1+ myeloid cells, B220+ B cells, and TCR+ T cells in flow cytometry (n = 3 in each). e–f Histology of BM (e) and spleens (f). g-h The numbers of megakaryocytes per high-power field (HPF) in BM (n = 3 in each) and spleens (n = 4—10). (*P < 0.05, **P < 0.01). [file 13045_2021_1064_MOESM2_ESM.pdf]

a

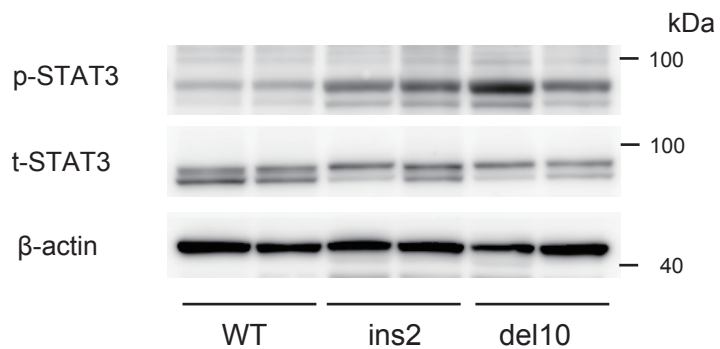

b

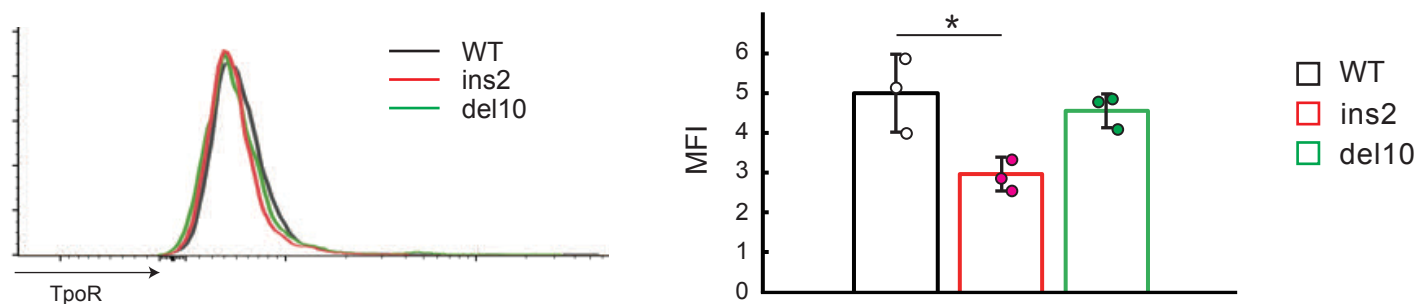

c

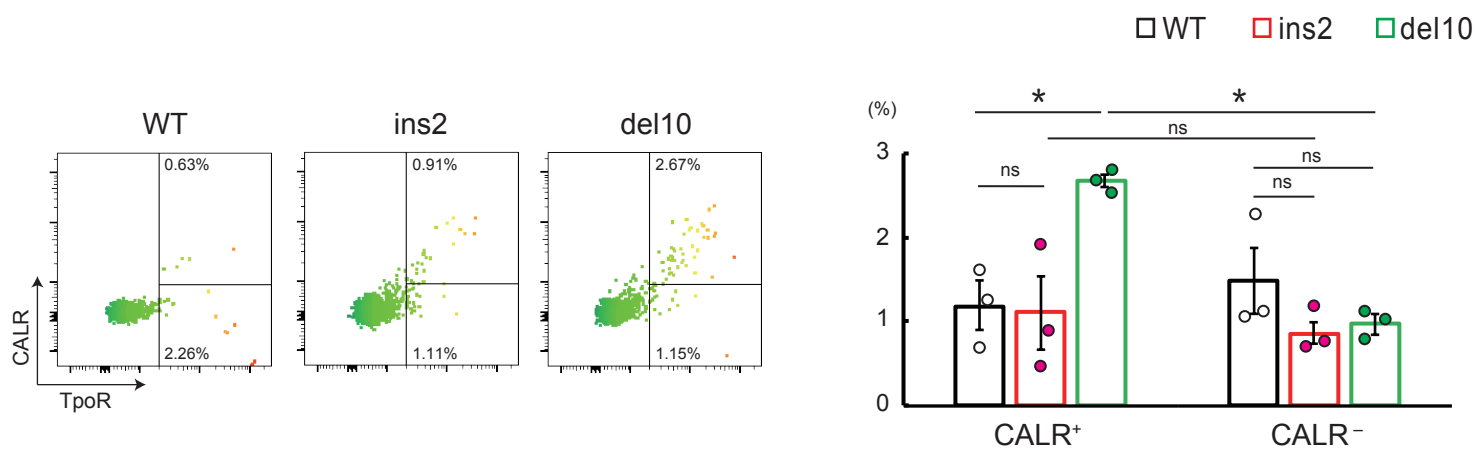

d

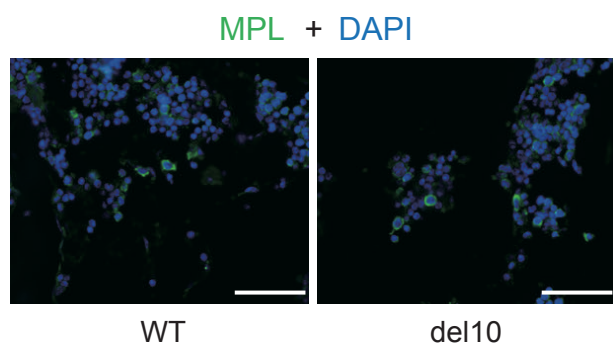

e

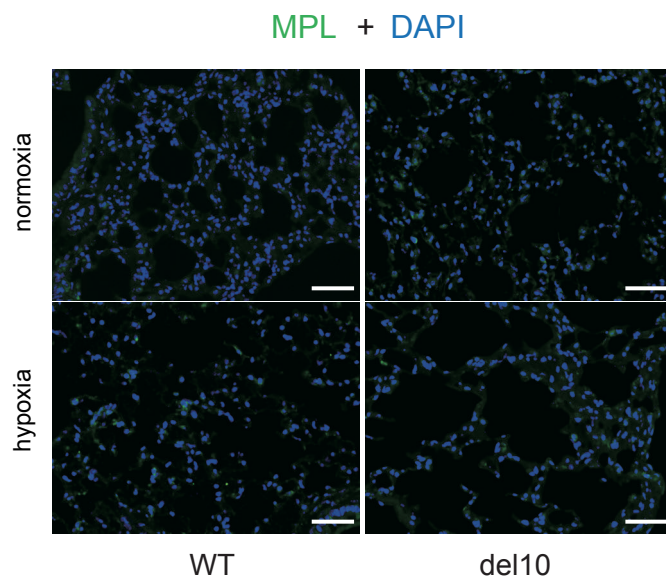

Supplement: Supplementary file 3 — Additional file 3. Fig. S3: Phosphorylation of STAT3 and expression of MPL, thrombopoietin receptor (TpoR). a Western blot of whole BM nuclear cells suspended in the absence of exogenous cytokines. b-c Flow cytometry gated with a lineage− fraction in BM cells. b Overall expression of cell-surface TpoR. Left: Histogram; right: mean fluorescence intensity (MFI). c Cell-surface expressions of TpoR and CALR. Left: heatmap plots; right: proportions of cell-surface TpoR+ cells in association with CALR expression (n = 3 in each experiment; *P < 0.05; ns: no significant difference). d Immunofluorescence for MPL in bone marrow. e Immunofluorescence for MPL in lung. d-e Scale bars, 50 µm. [file 13045_2021_1064_MOESM3_ESM.pdf]

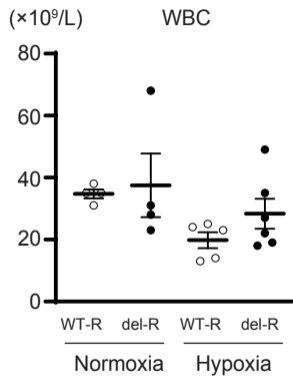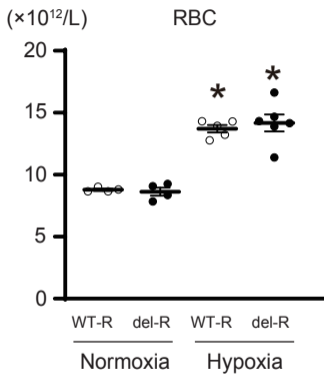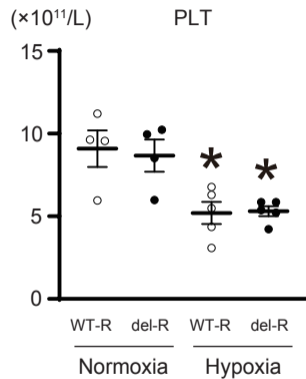

Supplement: Supplementary file 4 — Additional file 4. Fig. S4: Peripheral blood cell counts in the BMT recipients exposed to normoxia or chronic hypoxia for 3 weeks (n = 4–6). (*P < 0.05 versus the corresponding normoxia group). [file 13045_2021_1064_MOESM4_ESM.pdf]

*Edn1/Actb*

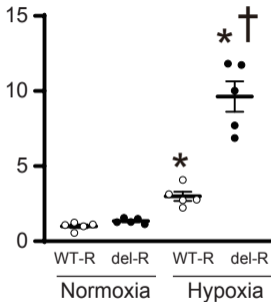

Supplement: Supplementary file 5 — Additional file 5. Fig. S5: Relative Edn1 mRNA expression levels in the lung (n = 5, each). The average value for WT-R mice under normoxia was set to 1. (*P < 0.05 versus the corresponding normoxia group, and †P versus the corresponding WT-R mice under chronic hypoxia) [file 13045_2021_1064_MOESM5_ESM.pdf]

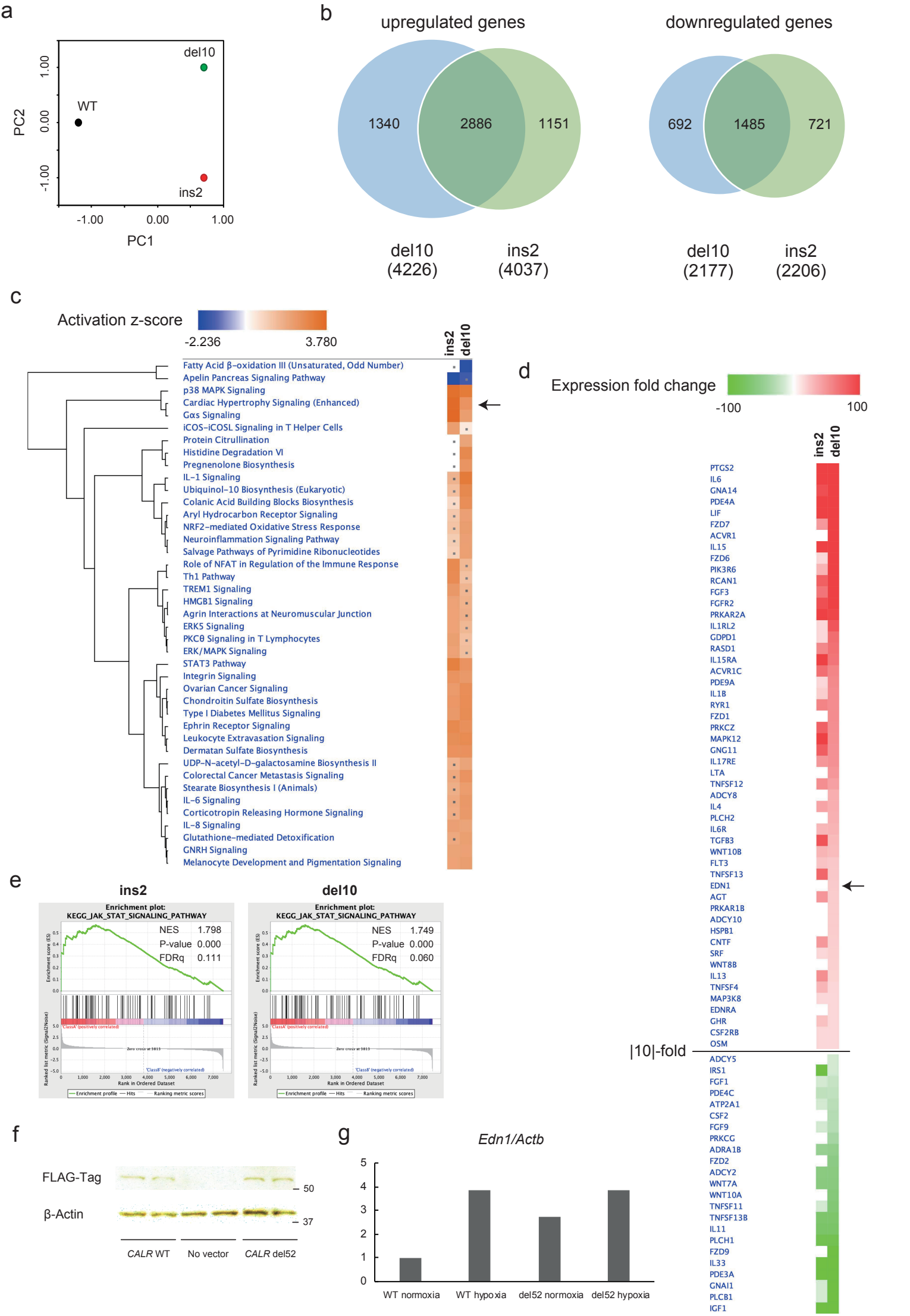

Supplement: Supplementary file 6 — Additional file 6. Fig. S6: Gene expressions. a-e RNA sequencing (RNAseq) in LSK (lineage–Sca1+c-Kit+) cells of an aliquot from 4 male mice of 3 months age in each sample from Calrins2/WT mice, Calrdel10/WT mice, and CalrWT/WT mice. a Principle component analysis. b Venn diagrams of upregulated and downregulated genes (> twofold) in LSK cells of Calrdel10/WT mice or Calrins2/WT mice relative to those of CalrWT/WT mice. c Pathway analysis by the Ingenuity Pathway Analysis software (Qiagen). All the pathways in the comparison analysis of canonical pathways with both Z score ≥|2| and p < 0.05 in at least one of the Calrins2/WT mice and Calrdel10/WT mice relative to CalrWT/WT mice are shown. • indicates the box which did not reach the level of Z score ≥|2| in the genotype shown. The allow indicates Cardiac Hypertrophy Signaling pathway upregulated in both Calrins2/WT mice and Calrdel10/WT mice. d Individual genes in the Cardiac Hypertrophy Signaling pathway. Differentially expressed genes ( >|10|-fold) in Calrdel10/WT mice relative to CalrWT/WT mice, including EDN1 that codes Endothelin-1 (allow), are shown. e Gene set enrichment analysis (GSEA) for the JAK-STAT pathway. NES indicates normalized enrichment score; FDRq, false discovery rate q value. f-g Introduction of FLAG-Tag-inserted human WT and del52 CALR constructs into a macrophage cell line, RAW 264.7. f Western blots. g The levels of Endothelin-1 mRNA (Edn1) were analyzed in RAW 264.7 cells introduced with CALR WT or del52 after incubation under normoxia (21% O2) or hypoxia (10% O2) for 24 h. Samples were taken from 3 wells for each experiment. Actb was used for normalization. The average value for cells introduced with WT CALR and incubated under normoxia was set to 1. [file 13045_2021_1064_MOESM6_ESM.pdf]
