# Supplementary material for: Myeloproliferative neoplasm-driving Calr frameshift promotes the development of pulmonary hypertension in mice
Source: J Hematol Oncol. 2021 Mar 30;14:52. doi: 10.1186/s13045-021-01064-8 (PMC8011226; doi:10.1186/s13045-021-01064-8)
Supplement: Supplementary file 7 — Additional file 7. Table S1: Frameshifts in CALR exon 9 on the COSMIC database in hematopoietic cancers. [file 13045_2021_1064_MOESM7_ESM.pdf]

Table S1. Frameshifts in CALR exon 9 on COSMIC database in hematopoietic cancers.

No.	Position	CDS Mutation	AA Mutation	Legacy Mutation ID	Count	Type
1	361	c.1080_1143delinsGGAAGAAGACAA	p.Q361Efs*52	COSM6022232	1	Complex - frameshift
2	363	c.1088delinsTTTGTC	p.E363Vfs*69	COSM5985668	3	Complex - frameshift
3	364	c.1089_1141del	p.E364Gfs*7	COSM5985674	1	Deletion - Frameshift
4	364	c.1090_1123del	p.E364Nfs*55	COSM3734989	2	Deletion - Frameshift
5	364	c.1090_1141del	p.E364Rfs*49	COSM5885137	1	Deletion - Frameshift
6	364	c.1091_1142del	p.E364Gfs*49	COSM1738057	108	Deletion - Frameshift
7	364	c.1092_1125del	p.E364Dfs*55	COSM1738333	3	Deletion - Frameshift
8	364	c.1092_1140delinsCCA	p.E364Dfs*51	COSM3355743	1	Complex - frameshift
9	365	c.1093_1138del	p.Q365Rfs*50	COSM3734990	4	Deletion - Frameshift
10	365	c.1094_1139del	p.Q365Rfs*50	COSM1738152	6	Deletion - Frameshift
11	366	c.1097_1130del	p.R366Kfs*53	COSM1738357	2	Deletion - Frameshift
12	367	c.1099_1132del	p.L367Rfs*52	COSM1738359	2	Deletion - Frameshift
13*	367	c.1099_1136del	p.L367Gfs*9	COSM9361241	2	Deletion - Frameshift
14	367	c.1099_1141del	p.L367Rfs*49	COSM5885131	1	Deletion - Frameshift
15	367	c.1099_1150del	p.L367Tfs*46	COSM1738055	1789	Deletion - Frameshift
16	367	c.1099_1153del	p.L367Rfs*45	COSM5885139	1	Deletion - Frameshift
17	367	c.1099_1156del	p.L367Rfs*44	COSM5885963	1	Deletion - Frameshift
18‡	367	c.1099_1159del	p.L367Mfs*43	COSM1738367	2	Deletion - Frameshift
19	367	c.1100_1133del	p.L367Rfs*52	COSM4745937	2	Deletion - Frameshift
20	367	c.1100_1134delinsA	p.L367Qfs*52	COSM1738339	2	Complex - frameshift
21	367	c.1100_1136delinsGGAGGT	p.L367Rfs*53	COSM5885968	1	Complex - frameshift
22	367	c.1100_1145del	p.L367Qfs*48	COSM1738150	35	Deletion - Frameshift
23	367	c.?	p.L367Qfs*?	COSM5574296	2	Deletion - Frameshift
24	367	c.?	p.L367Xfs*46	COSM3355748	22	Deletion - Frameshift
25	368	c.1101_1134del	p.K368Rfs*51	COSM1738368	2	Deletion - Frameshift
26	368	c.1101_1146del	p.K368Rfs*47	COSM5885962	1	Deletion - Frameshift
27	368	c.1101_1152del	p.K368Rfs*45	COSM1738334	1	Deletion - Frameshift
28	368	c.1101_1161del	p.K368Mfs*42	COSM1738360	1	Deletion - Frameshift
29	368	c.1102_1137delinsCA	p.K368Qfs*51	COSM1738157	2	Complex - frameshift
30	368	c.1102_1137delinsGA	p.K368Efs*51	COSM3355754	2	Complex - frameshift
31	368	c.1103_1136del	p.K368Rfs*51	COSM1738151	23	Deletion - Frameshift
32	368	c.1103_1139del	p.K368Rfs*50	COSM5885104	1	Deletion - Frameshift

33	368	c.1103_1145delinsGGAGGAGGG	p.K368Rfs*51	COSM3355744	1	Complex - frameshift
34	368	c.1103_1148del	p.K368Rfs*47	COSM3355766	3	Deletion - Frameshift
35	368	c.1103_1154del	p.K368Rfs*45	COSM1738343	9	Deletion - Frameshift
36	369	c.1105_1138del	p.E369Rfs*50	COSM1738153	8	Deletion - Frameshift
37	369	c.1105_1147delinsAGGAGGCAA	p.E369Rfs*50	COSM5885974	1	Complex - frameshift
38	369	c.1105_1148delinsAGGAGGCAGT	p.E369Rfs*50	COSM3355745	1	Complex - frameshift
39	369	c.1105_1156del	p.E369Rfs*44	COSM1738158	2	Deletion - Frameshift
40	370	c.1108_1138del	p.E370Rfs*50	COSM5731855	1	Deletion - Frameshift
41	370	c.1108_1141del	p.E370Rfs*49	COSM3733223	1	Deletion - Frameshift
42	370	c.1108_1144del	p.E370Qfs*48	COSM1738361	1	Deletion - Frameshift
43	370	c.1108_1177del	p.E370Rfs*37	COSM1738364	1	Deletion - Frameshift
44	370	c.1109_1160del	p.E370Vfs*43	COSM6022228	1	Deletion - Frameshift
45	371	c.1111_1141del	p.E371Rfs*49	COSM1738349	5	Deletion - Frameshift
46	371	c.1111_1142delinsT	p.E371Wfs*49	COSM5731860	1	Complex - frameshift
47	371	c.1111_1147del	p.E371Rfs*47	COSM1738335	4	Deletion - Frameshift
48	371	c.1111_1150del	p.E371Tfs*46	COSM9114826	1	Deletion - Frameshift
49	371	c.1112_1142del	p.E371Gfs*49	COSM5703407	1	Deletion - Frameshift
50	371	c.1113_1125delinsTTGTCT	p.E371Dfs*57	COSM2270994	1	Complex - frameshift
51	372	c.1114_1144del	p.E372Qfs*48	COSM3734991	2	Deletion - Frameshift
52	373	c.1116_1146del	p.D373Rfs*47	COSM2704902	6	Deletion - Frameshift
53	373	c.1116del	p.D373Tfs*57	COSM6506477	1	Deletion - Frameshift
54	373	c.1118_1136del	p.D373Gfs*51	COSM1738159	2	Deletion - Frameshift
55	373	c.1118_1145delinsCGTTTA	p.D373Afs*50	COSM1738160	2	Complex - frameshift
56	373	c.?	p.D373Xfs*?	COSM9312386	1	Deletion - Frameshift
57	374	c.1120_1125delinsTCTTGTCT	p.K374Sfs*57	COSM5703410	1	Complex - frameshift
58	374	c.1120_1125delinsTGCGT	p.K374Cfs*56	COSM3355758	2	Complex - frameshift
59	374	c.1120_1126delinsTACGTA	p.K374Yfs*56	COSM3355765	1	Complex - frameshift
60	374	c.1120_1126delinsTTCTTGTCTTCTTGTCTT	p.K374Ffs*60	COSM5985670	1	Complex - frameshift
61	374	c.1120_1131delinsTGCGT	p.K374Cfs*54	COSM1738344	1	Complex - frameshift
62	374	c.1120_1140delinsTCTTGTCT	p.K374Sfs*52	COSM5885136	1	Complex - frameshift
63	374	c.1121_1139del	p.K374Rfs*50	COSM1738345	2	Deletion - Frameshift
64	374	c.1121_1142del	p.K374Rfs*49	COSM9114827	3	Deletion - Frameshift
65	374	c.1121_1148del	p.K374Rfs*47	COSM3734992	1	Deletion - Frameshift
66	374	c.1122_1123delinsTTGT	p.K374Nfs*57	COSM5703409	1	Complex - frameshift

67	374	c.1122_1125del	p.K374Nfs*55	COSM1738328	9	Deletion - Frameshift
**68**	**375**	**c.1122_1141delinsA**	**p.K375Rfs*49**	**COSM1738346**	**1**	**Complex - frameshift**
**69**	**375**	**c.1122del**	**p.K375Nfs*55**	**COSM1738350**	**2**	**Deletion - Frameshift**
**70**	**375**	**c.1123_1125delinsTGTTT**	**p.K375Cfs*56**	**COSM1738351**	**2**	**Complex - frameshift**
**71**	**375**	**c.1123_1125delinsTTTTGTTT**	**p.K375Ffs*57**	**COSM5885965**	**1**	**Complex - frameshift**
**72**	**375**	**c.1123_1133del**	**p.K375Gfs*10**	**COSM9264991**	**2**	**Deletion - Frameshift**
**73**	**375**	**c.1123_1144del**	**p.K375Qfs*48**	**COSM5703406**	**1**	**Deletion - Frameshift**
**74**	**375**	**c.1124_1133del**	**p.K375Rfs*52**	**COSM1738366**	**2**	**Deletion - Frameshift**
**75**	**375**	**c.1124_1136del**	**p.K375Rfs*51**	**COSM6022233**	**1**	**Deletion - Frameshift**
**76**	**375**	**c.1124_1142del**	**p.K375Rfs*49**	**COSM1738337**	**4**	**Deletion - Frameshift**
77	375	c.1125del	p.K375Nfs*55	COSM5885245	1	Deletion - Frameshift
78	376	c.1125_1126insTTCTTAGTGCT	p.R376Ffs*58	COSM6022230	1	Insertion - Frameshift
79	376	c.1126_1131delinsTGCGT	p.R376Cfs*54	COSM5985672	1	Complex - frameshift
80	376	c.1126_1148del	p.R376Gfs*5	COSM6852891	1	Deletion - Frameshift
81	376	c.1127_1129delinsTTTGC	p.R376Lfs*55	COSM1738363	1	Complex - frameshift
82	376	c.1127_1132delinsTTTGC	p.R376Lfs*54	COSM5885105	2	Complex - frameshift
83	376	c.1127_1145del	p.R376Qfs*48	COSM4745934	3	Deletion - Frameshift
84	377	c.1125_1146del	p.K377Tfs*46	COSM3734993	2	Deletion - Frameshift
85	377	c.1129_1135delinsCTTTGCGTA	p.K377Lfs*54	COSM5885110	1	Complex - frameshift
86	377	c.1129_1139del	p.K377Gfs*8	COSM5037721	1	Deletion - Frameshift
87	377	c.1129_1139delinsCTCTGCCTCC	p.K377Lfs*53	COSM5037722	1	Complex - frameshift
88†	377	c.1130_1139del	p.K377Rfs*50	COSM3355747	1	Deletion - Frameshift
89	377	c.1130_1154delinsTCCATCCTTGTC	p.K377Ifs*49	COSM5885106	1	Complex - frameshift
**90**	**378**	**c.1132_1153del**	**p.E378Rfs*45**	**COSM1738329**	**4**	**Deletion - Frameshift**
**91**	**378**	**c.?**	**p.E378Xfs*45**	**COSM6834816**	**1**	**Deletion - Frameshift**
92	379	c.1135_1147del	p.E379Rfs*47	COSM4745635	1	Deletion - Frameshift
93	379	c.1135_1152delinsCCTCCTCTTTGTCT	p.E379Pfs*50	COSM1738352	1	Complex - frameshift
94	379	c.1135del	p.E379Rfs*51	COSM9285102	1	Deletion - Frameshift
95	379	c.1136del	p.E379Gfs*51	COSM9264994	2	Deletion - Frameshift
96	379	c.1137_1154delinsCCATCCTTGTC	p.E379Dfs*49	COSM1738353	1	Complex - frameshift
97	380	c.1136_1137dup	p.E380Rfs*51	COSM4387491	1	Insertion - Frameshift
98	380	c.1138_1212delinsAGGAGGCAGAGGACAAGGAGG ATGATGAGGACAAAGATGAGGATGAGGAGGATGA GGAGGACAAGGAGGAAGATGA	p.E380Rfs*62	COSM5968696	1	Complex - frameshift
99	380	c.1139_1140insTC	p.E380Dfs*51	COSM3355767	2	Insertion - Frameshift

100	380	c.1139_1215delinsCAAGGAGGATGATGAGGACAAAGATGAGGATGAGGAGGATGAGGAGGACAAGGAGGAAGTTGA	p.E380Afs*57	COSM7338412	1	Complex - frameshift
101	380	c.1139del	p.E380Gfs*50	COSM5885111	2	Deletion - Frameshift
102	381	c.1141del	p.E381Rfs*49	COSM1738154	4	Deletion - Frameshift
103	381	c.1142_1151delinsCGGCATGTC	p.E381Afs*49	COSM5703411	1	Complex - frameshift
104	381	c.1143_1154delinsTCCTTGTC	p.E381Dfs*48	COSM1738362	1	Complex - frameshift
105	382	c.1144_1220delinsCAGAGGACAAGGAGGATGATGAGGACAAAGATGAGGATGAGGAGGATGAGGAGGACAAGGAGGAAGATG	p.A382Qfs*57	COSM3355735	1	Complex - frameshift
106	383	c.1145_1146insGACGC	p.E383Tfs*49	COSM5703408	1	Insertion - Frameshift
107	383	c.1147_1151del	p.E383Qfs*4	COSM9180538	1	Deletion - Frameshift
108	383	c.1147_1154delinsTGTC	p.E383Cfs*46	COSM5037719	2	Complex - frameshift
109	383	c.1148_1149insTCCTTGTC	p.E383Dfs*50	COSM5885135	1	Insertion - Frameshift
110	383	c.1148_1154delinsGAC	p.E383Gfs*46	COSM4745936	1	Complex - frameshift
111	384	c.1145_1149dup	p.D384Qfs*48	COSM9180539	1	Insertion - Frameshift
112	384	c.1150_1151ins52	p.D384fs*?	COSM5715393	1	Insertion - Frameshift
113	384	c.1150_1153delinsTTGTCCTCCTCCTGCCTC	p.D384Lfs*51	COSM5885112	1	Complex - frameshift
114	384	c.1150_1154delinsTGTC	p.D384Cfs*46	COSM6022229	1	Complex - frameshift
115	384	c.1151_1154delinsGCAATTGTC	p.D384Gfs*48	COSM3355746	2	Complex - frameshift
116	384	c.1151_1154delinsTATGTC	p.D384Vfs*47	COSM3355768	1	Complex - frameshift
117	384	c.1151_1154delinsTTTGTC	p.D384Vfs*47	COSM5037720	1	Complex - frameshift
118	385	c.1153_1154delinsTCTTGTC	p.K385Sfs*47	COSM1738365	1	Complex - frameshift
119	385	c.1153_1154delinsTGTC	p.K385Cfs*46	COSM1738330	4	Complex - frameshift
120	385	c.1153_1154insTCTGT	p.K385Ifs*47	COSM4745935	1	Insertion - Frameshift
121	385	c.1154_1155insTTGTC	p.K385Nfs*47	COSM1738056	1129	Insertion - Frameshift
122	385	c.1154del	p.K385Rfs*45	COSM9180541	3	Deletion - Frameshift
123	385	c.1154delinsCTTGTC	p.K385Tfs*47	COSM1738331	6	Complex - frameshift
124	385	c.1154delinsTATGTC	p.K385Ifs*47	COSM5985669	1	Complex - frameshift
125	385	c.1154delinsTCTGTC	p.K385Ifs*47	COSM3734994	1	Complex - frameshift
126	385	c.1154delinsTGTGTC	p.K385Mfs*47	COSM1738155	6	Complex - frameshift
127	385	c.1154delinsTTTGTC	p.K385Ifs*47	COSM1738356	3	Complex - frameshift
128	385	c.?	p.K385Xfs*47	COSM3355749	27	Insertion - Frameshift
129	386	c.1154_1155insATGTC	p.E386Cfs*46	COSM1738355	6	Insertion - Frameshift
130	386	c.1155_1156insTGTCG	p.E386Cfs*46	COSM1738332	11	Insertion - Frameshift
131	387	c.1153_1157dup	p.D387Rfs*45	COSM5885138	1	Insertion - Frameshift
132	387	c.1157_1158dup	p.D387Rfs*44	COSM9264993	2	Insertion - Frameshift

133	387	c.1159_1177del	p.D387Rfs*37	COSM5967353	1	Deletion - Frameshift
134	387	c.1159del	p.D387Mfs*43	COSM5885113	1	Deletion - Frameshift
135	388	c.1162del	p.D388Mfs*42	COSM9226088	1	Deletion - Frameshift
136	404	c.1211_1217del	p.D404Gfs*24	COSM5574343	2	Deletion - Frameshift
137	405	c.1214del	p.E405Gfs*25	COSM5753346	1	Deletion - Frameshift
138	413	c.1227_1231del	p.A413Gfs*27	COSM9269787	2	Deletion - Frameshift

We also searched frameshifts in *CALR* exon 9 in patients with myeloproliferative neoplasms (MPNs) in a public database, COSMIC (Catalogue Of Somatic Mutations In Cancer, v92, released on 27-Aug-20, https://cancer.sanger.ac.uk/cosmic), filtering the position, mutation types, and organ (hematopoietic and lymphoid), followed by identification of diagnosis. Of 138 mutations, 136 (98.6%) were associated with Philadelphia-negative MPNs, although the subtypes were not available in several cases. Nos.13 (*) and 88 (†) were detected only in CML and MDS, respectively. In No.18 (‡), one of 2 cases was a patient with MDS. In all other mutations, vast majority of cases were patients with ET, PMF or secondary acute leukemia from MPNs. Mutations starting from codon 375 or 378, corresponding to del10 or ins2 in murine *Calr*, are indicated with bold letters. No.74, c.1124_1133del (p.K375Rfs*52), which exactly matches murine *Calr* del10, is indicated in red and bold letters.
