# Supplementary material for: Myeloproliferative neoplasm-driving Calr frameshift promotes the development of pulmonary hypertension in mice
Source: J Hematol Oncol. 2021 Mar 30;14:52. doi: 10.1186/s13045-021-01064-8 (PMC8011226; doi:10.1186/s13045-021-01064-8)
Supplement: Supplementary file 8 — Additional file 8. Table S2: Oligonucleotides used in this study. [file 13045_2021_1064_MOESM8_ESM.pdf]

Table S2. Oligonucleotides used in this study.

Description	Sequence (5' → 3')	Experiment
**crRNA***	**AGAGGACAAGAAGCGUAAAGG**uuuuaGAgcuaugcuguuuUG	CRISPR-Cas9
**tracrRNA**	aaacagcauagcAAGUuaaaaUaaggcuaguccguuaucaacUUGAA aaaGUGgcaccgaGucggugcUUUuuuU	CRISPR-Cas9
**Donor oligo 1†**	GGTTGGTCCTCACAGGCTGCAGAGAAGCAGATGAAGGAC AAGCAGGATGAGGAGCAGAGGATAAAGAGGATGATGATG ACAGAGATGAAGATGAGGACGAAGAAGATGAGAAGGAGG AAG	CRISPR-Cas9
**Donor oligo 2‡**	AAGAGGACAAGAAGCGTAAAGAGGAAGAAGAAGCTGAGG ATAAAGAGGATTTGTCGATGATGACAGAGATGAAGATGA GGACGAAGAAGATGAGAAGGAGGAAGA	CRISPR-Cas9
***Calr*-Fw**	GGAGGCAGGGGAACAAAATCA	PCR, Sanger sequence
***Calr*-Rv**	TCAAAGACTAGGAAAGGTGGGAAT	PCR, Sanger sequence
***Calr*-qPCR-Fw**	TCATCATCCTCTTTATCCTCAGCTT	qPCR genotyping
***Calr*-qPCR-Rv**	TGAGGAGCAGAGGCTTAAGGAA	qPCR genotyping
***Calr*-probe (wild type)**	VIC-TCTTCCTCTTTACGCTTC-MGB	qPCR genotyping
***Calr*-probe (ins2)**	FAM-TTCCTCTTTTTACGCTTC-MGB	qPCR genotyping
***Calr*-probe (del10)**	FAM-TCCTCTTGTCCTCTTC-MGB	qPCR genotyping
***Edn1*-Fw**	CTACGAAGGTTGGAGGCC	qRT-PCR
***Edn1*-Rv**	CGGTTGTGCGTCAACTTCTG	qRT-PCR
***Actb*-Fw**	CATCCGTAAAGACCTCTATGCCAAC	qRT-PCR
***Actb*-Rev**	ATGGAGCCACCGATCCACA	qRT-PCR

*: Bold font indicates protospacer adjustment motif site from the sequence in exon 9 of murine *Calr* mRNA (NM_007591.3) determined using the CRISPR direct (https://crispr.dbcls.jp). † and ‡ were oligodeoxynucleotide containing putative sequences for mutant murine *Calr*, del52 and ins5, respectively. Fw, forward; Rv, reverse.
