# Supplementary material for: Myeloproliferative neoplasm-driving Calr frameshift promotes the development of pulmonary hypertension in mice
Source: J Hematol Oncol. 2021 Mar 30;14:52. doi: 10.1186/s13045-021-01064-8 (PMC8011226; doi:10.1186/s13045-021-01064-8)
Supplement: Supplementary file 9 — Additional file 9. Table S3: Antibodies used in this study. [file 13045_2021_1064_MOESM9_ESM.pdf]

Table S3. Antibodies used in this study.

Name	Experiment	Vendor	Cat. Number
**Ter-119 (FITC)**	Flow cytometry	Thermo Fisher Scientific	11-5921-81
**Gr-1 (APC)**	Flow cytometry	Thermo Fisher Scientific	17-9668-80
**TCRβ (PerCp/Cy5.5)**	Flow cytometry	Thermo Fisher Scientific	45-5961-80
**CD45R (eFluor450)**	Flow cytometry	Thermo Fisher Scientific	48-0452-80
**MPL (Alexa Fluora488)**	Flow cytometry	Bioss	Bs-10362R-A488
**Lineage cocktail (PerCP/Cy5.5)**	Flow cytometry	BD	561317
**CALR-PE (N terminal)***	Flow cytometry	Abcam	ab209577
**Sca-1 (Ly6A/E-APC)**	Flow cytometry	Thermo Fisher Scientific	17-5981-81
**CD117 (cKit-PE)**	Flow cytometry	Thermo Fisher Scientific	12-1171-81
**CD41**	IHC	Abcam	ab134141
**α-smooth muscle actin (α-SMA)**	IHC	Cell Signaling Technology	19245
**F4/80**	IHC	Santa Cruz Biotechnology	sc-52664
**GFP**	IHC	Novus Biologicals	NBP2-22111
**Anti-rabbit IgG (Alexa Flour 647)**	IHC	Cell Signaling Technology	4414S
**CALR (N terminal)†**	Western	Abcam	ab94935
**CALR (C terminal)‡**	Western	Abcam	ab2908
**Endothelin-1**	Western	Abcam	ab2786
**Phospho-STAT3**	Western	Cell Signaling Technology	9145P
**STAT3**	Western	Cell Signaling Technology	8768P
**FLAG-Tag**	Western	Cell Signaling Technology	14793
**β-actin**	Western	Proteintech	20536-1-AP

*: PE-conjugated antibody immunized with synthetic peptide within N-terminal side of amino acid (aa50-150) of human CALR. IHC indicates immunohistochemistry. † and ‡ are antibodies specific for N-terminal (aa24-43) and C-terminal (aa399-414) sides of human CALR, respectively.
