# Supplementary material for: Myeloproliferative neoplasm-driving Calr frameshift promotes the development of pulmonary hypertension in mice
Source: J Hematol Oncol. 2021 Mar 30;14:52. doi: 10.1186/s13045-021-01064-8 (PMC8011226; doi:10.1186/s13045-021-01064-8)
Supplement: Supplementary file 10 — Additional file 10: Supplementary methods, results, and references. [file 13045_2021_1064_MOESM10_ESM.pdf]

Supplementary methods, results and references

Methods

Genome editing and mice

For genome editing, we searched PAM (protospacer adjustment motif) sites in exon 9 of murine *Calr* mRNA (NM_007591.3) using CRISPR direct (https://crispr.dbcls.jp), and identified a sequence for CRISPR RNA (crRNA) with few off-targets (Table S2). The CRISPR RNA and a trans-acting CRISPR RNA (tracrRNA) were annealed using the Alt-R™ CRISPR-Cas9 system (IDT, Coralville, IA, USA) and then mixed with Cas9 protein to form Cas9-RNP complex in Opti-MEM I (Thermo Fisher Scientific, Waltham, MA, USA). The Cas9-RNP complexes and donor single-stranded synthetic oligodeoxynucleotides of putative mutant *Calr* exon 9 (Table S2) were introduced into fertilized eggs from C57BL/6 J mice by electroporation, as described previously [1]. The manipulated eggs were then cultured in mWM medium (COSMO Bio, Tokyo, Japan) at 37 °C and 5% CO_2 overnight. The surviving 2-cell-stage embryos were transferred to the oviducts of pseudopregnant mice. We screened pups by PCR followed by Sanger sequencing of genomic DNA derived from tail tips, and eventually obtained knock-in mice with *del10* and with *ins2* as founders, instead of del52 or ins5. These founders were crossed with *WT* C57BL6/J mice, and subsequent generations were screened by real-time PCR with probes specific for *WT*, *ins2* and *del10 Calr*. Third or later generations of heterozygous KI mice ($Calr^{del10/WT}$ mice and $Calr^{ins2/WT}$ mice), and WT littermates ($Calr^{WT/WT}$ mice) were used for experiments. Then, CAG-EGFP reporter mice with a C57BL/J background were purchased from the Japan SLC (Shizuoka, Japan). $Calr^{del10/WT}$ mice were crossed with CAG-EGFP mice to generate $Calr^{del10/WT}$/CAG-EGFP mice. All mice were analyzed at 3 months after birth unless otherwise specified. Sex was matched among genotypes in each experiment.

Studies based on on-line software and database

We assessed isoelectric point (pI), the pH at which a molecule is electrically neutral, of

peptides from codon A352 to the C terminus coded by WT or frameshifted murine *Calr* exon 9, using Protein Calculator v3.4 (http://protcalc.sourceforge.net) [2]. Alignment of the same portion was analyzed by EMBOSS Needle (EMBL-EBI, https://www.ebi.ac.uk/Tools/psa/emboss_needle/) [3]. We also searched frameshifts in *CALR* exon 9 in patients with myeloproliferative neoplasms (MPNs) in a public database, COSMIC (Catalogue Of Somatic Mutations In Cancer, v92, released on 27-Aug-20, https://cancer.sanger.ac.uk/cosmic). The subtype of MPN was not available in some COSMIC data.

Peripheral blood and bone marrow cells

Peripheral blood (PB) was collected from tail veins and complete blood count was determined using a pocH-100i (Sysmex, Kobe, Japan). Bone marrow (BM) cells were collected by grinding bones and counted by a TC10 (BioRad, Hercules, CA, USA). BM cells were used for flow cytometry, Western blotting, BM transplantation (BMT) and preparation of macrophages.

Flow cytometry

BM cells were stained with fluorescent-conjugated antibodies (Table S3) and assessed by flow cytometry using a FACSCanto II (BD, Franklin Lakes, New Jersey, USA).

Western blotting

BM nuclear cells, lungs, and cultured cells were homogenized in lysis buffer, and the protein concentration was determined in each sample using a Piece BCA Protein Assay Kit (Thermo Fischer Scientific). Aliquots of the protein samples were subjected to SDS-polyacrylamide gel electrophoresis, transferred onto polyvinylidene difluoride membranes (Merck Millipore, Burlington, MA, USA), blocked with 5% bovine serum albumin (FUJIFILM Wako, Osaka, Japan), and probed with antibodies (Table S3). The immunoreactive bands were visualized by an Amersham ECL and the signals were detected with an Amersham Imager 600 RGB (GE Healthcare Life Sciences, MA, USA). The results were evaluated by densiometric

analysis of the lytic areas obtained from the gelatin electrophoresis using ImageJ software (National Institutes of Health, Bethesda, MD, USA).

Histopathology

Samples were fixed in 4% paraformaldehyde solution and sectioned to 3 μm thickness. The paraffin sections were stained with hematoxylin-eosin (HE) or used for immunostaining with a CD41, α-smooth muscle actin (α-SMA), F4/80, or GFP antibody (Table S3) followed by 0.02% 3,3-diaminobenzidine-4HCl (Dojindo, Kumamoto, Japan) and counterstained with hematoxylin. Frozen lung tissues were embedded in O.C.T. compound (Sakura Finetek Japan, Tokyo, Japan) and were sectioned to 12 μm thickness using a cryostat (CryoStar NX70, Thermo Fisher Scientific, MA, USA). The O.C.T.-embedded sections were used for immunohistochemical staining with the α-SMA, F4/80, or GFP antibody followed by the appropriate secondary antibody of goat anti-rabbit IgG, and them mounted with DAPI containing mounting media (Fluoro-Gel II, Electron Microscopy Sciences, PA, USA). All images were acquired by a microscope (BZ-X700, KEYENCE, Osaka, Japan).

Bone marrow transplantation

As recipients, male C57BL/J mice aged between 8 and 12 weeks (body weights 20 to 30 g) were lethally irradiated (9.0 Gy) 24 hours before BMT. BM cells were harvested from the femurs and tibiae of donor mice. The cells were washed with phosphate-buffered saline (PBS) and 5.0 x 10^6 whole BM cells were injected into the C57BL/J mice via the tail vein[4]. Four weeks after BMT, PB was obtained from recipients to determine CBC and allele frequency of del10 *Calr.* Genomic DNA was isolated using Quickgene-Mini80 (KURABO, Osaka, Japan) and QuickGene DNA whole blood kit (KURABO), and the allele frequency of del10 *Calr* was evaluated by quantitative PCR with THUNDERBIRD Probe qPCR Mix (TOYOBO, Osaka, Japan) and probes specific for WT and del10 *Calr* (Table S2) using a QuantStudio3 real-time PCR system (Thermo Fisher Scientific, MA, USA). Delta Ct (Ct_{donor}-Ct_{total}) was calculated to estimate the allele frequencies of del10 *Calr* in the recipients.

Hypoxic exposure

The BMT recipient mice were exposed to normoxia (21% O2) or hypoxia (10% O2) for 3 weeks [5]. The chronic hypoxic condition was maintained by a mixture of air and nitrogen in a ventilated chamber system (Teijin Ltd., Osaka, Japan). The ventilated chamber system was kept closed except for supplying food and water and cleaning the cages twice a week.

Study of right ventricular pressure and right ventricular hypertrophy

After chronic exposure to normoxia or hypoxia, the BMT recipient mice were anesthetized by intraperitoneal injection of 2,2,2-tribromo-ethanol (0.25 mg/g per body weight). Right ventricular pressure was continuously measured by a 1.2F micromanometer catheter (Transonic Science Inc., NY, USA), inserted from the right jugular vein. The right ventricular systolic pressure (RVSP) was blindly assessed using LabScribe3 software (iWork Systems, Inc., NH, USA) and was averaged over 10 sequential beats. The right ventricle was dissected from the left ventricle including the septum for analyzing right ventricular hypertrophy. Right ventricle/left ventricle + septum weight ratio (RV/LV+IVS) was calculated.

Quantitative reverse-transcription PCR (qRT-PCR)

Total RNA from the lungs and cultured cells were extracted using RNeasy Fibrous Tissue Mini Kit (QIAGEN, Hilden, Germany) and Trizol reagent (Thermo Fischer Scientific), respectively. Complementary DNA was synthesized using ReverTra Ace qPCR RT Kit (TOYOBO). The levels of mRNA expression for *Edn1* and *Actb* were evaluated by qRT-PCR using THUNDERBIRD SYBR qPCR Mix (TOYOBO) in the CFX Connect Real-time PCR System (Bio-RAD). *Edn1* values were normalized to *Actb* and expressed as a fold increase of the control group. Primer sequences are described in Table S2.

Concentrations of total cholesterol

Concentrations of total cholesterol in serum samples were assessed by the enzyme-linked immuno-sorbent assay using a LabAssay cholesterol (FUJIFILM Wako).

Bone marrow-derived macrophages and a macrophage cell line

To prepare BM-derived macrophages, mononuclear cells (MNCs) were collected from whole BM cell suspension of *Calr*$^{del10/WT}$ mice and *Calr*$^{WT/WT}$ mice by density gradient centrifugation at 500 g for 40 min at room temperature using Lymphocyte Separation Media (Promo Cell, Heidelberg, Germany). On a 24-well cell culture plate (662160, CELLSTAR, Greiner Bio-One, Kremsmünster, Austria) for total RNA extraction, 2 x 10^5 of the separated MNCs per well, and on a 6-well cell culture plate (353046, Falcon plates, Corning, AZ, USA) for protein extraction, 80 x 10^5 of the separated MNCs per well were cultured in DMEM (FUJIFILM Wako Pure Chemical Corporation, Osaka, Japan) containing 10% fetal bovine serum (FBS), 100 IU/mL of penicillin, 100 mg/mL of streptomycin and 10 ng/mL of macrophage-colony stimulating factor (416-ML, R&D systems, MN, USA) for 6 days at 37°C in the 5% CO_2, as described previously [6,7]. The BM-derived macrophages were treated with 0.05 μg/mL of lipopolysaccharide (LPS) for 0, 12, and 24 hours. To determine the purity of the macrophages, the adherent cells were seeded in a 4-well chamber slide (Thermo Fisher Scientific) and fixed by 4% paraformaldehyde and stained with a F4/80 antibody followed by the appropriate secondary antibodies, and then mounted with DAPI containing mounting media. A macrophage cell line, RAW 264.7 was cultured in RPMI1640 (FUJIFILM Wako Pure Chemical Corporation, Osaka, Japan) containing 10% FBS, 100 IU/mL of penicillin, and 100 mg/mL of streptomycin. For cell culture under the hypoxic condition, oxygen concentration was set to 10% at 37°C in the 5% CO_2.

RNA sequencing (RNAseq)

Total RNA was extracted from BM lineage$^-$Sca1$^+$Kit$^+$ (LSK) cells enriched with hematopoietic progenitor cells in an aliquot from 4 male mice of 3 months age in each sample using a miRNeasy Micro Kit (Qiagen, Hilden, Germany). The integrity and quantity of the total RNA was measured with an Agilent 2100 Bioanalyzer RNA 6000 Pico Kit (Agilent, Santa Clara, CA, USA). From the total RNA obtained from each sample, 5 ng was used for a sequencing library construction using NEBNext Ultra II Directional RNA Library Prep Kit and NEBNext

rRNA Depletion Kit (New England BioLabs, Beverly, MA, USA). The quality of the libraries was assessed with an Agilent 2200 TapeStation High Sensitivity D1000 (Agilent). The pooled libraries of the samples were sequenced using a NextSeq 500 (Illumina, San Diego, CA, USA) in 75-bp single-end reads. Sequencing adaptors, low quality reads, and bases were trimmed using Trimmomatic-0.32 [8]. The sequence reads were aligned to the mouse reference genome (mm10) using Tophat 2.1.1 (bowtie2-2.2.3) [9]. Files of the gene model annotations and known transcripts were obtained from iGenomes (Illumina, http://support.illumina.com/sequencing/sequencing_software/igenome.html) for whole transcriptome alignment with Tophat. The aligned reads were subjected to downstream analyses using StrandNGS 3.2 (Agilent). The read counts allocated for each gene and transcript (RefSeq Genes 2013.04.01) were quantified using a Trimmed Mean of M-value [10]. The RNAseq data have been deposited in the Gene Expression Omnibus database (GSE152482). The data were also subjected to Ingenuity Pathway Analysis (QIAGEN) and Gene Set Enrichment Analysis (GSEA, Broad Institute, Cambridge, MA, USA).

Human *CALR* construct

Human *CALR* cDNA (MGC clone ID: 3898550), inserted into pCMV-SPORT6, was purchased from DNAFORM (Yokohama, Japan). *CALR*-del52 mutation [11,12] was generated using a KOD Plus Mutagenesis kit (Toyobo, Osaka, Japan). FLAG-Tag-introduced *WT* or *del52 CALR* cDNA (pCMV-FLAG *CALR* WT or pCMV-FLAG *CALR* del52, respectively) was transfected to RAW 264.7 cells in a 24-well plate using a GenomOne-GX (Ishihara Sangyo Kaisha, Osaka, Japan) according to the manufacturer's protocol, and the expression was confirmed by Western blotting with an anti-FLAG Tag antibody.

Statistical analysis

Data are expressed as mean ± SEM in entire manuscript including supplementary figures. The statistical significance of differences was analyzed using the unpaired Student's t-test for parametric continuous variables. The parameters for more than 2 groups were evaluated by one-way analysis of variance (ANOVA) followed by multiple comparisons with the Turkey

post-hoc test. A value of $P<0.05$ was considered statistically significant. Statistical analyses were performed using the Statistical Package for Social Sciences version 26 software (SPSS Inc., Chicago, IL, USA).

Supplementary results

Characterization of murine *Calr* mutations

Genome editing with the CRISPR-Cas9 method yielded 2 lines of knock-in mice with frameshifts in murine *Calr*, ins2 (CALR p.E378Kfs*53) and del10 (CALR p.K375Rfs*52). The C domain of WT CALR protein acts as a calcium buffering domain and is mainly encoded by exon 9, in which negatively charged amino acids are dominant in both human and mouse. As with CALR mutations in human MPNs, murine CALR ins2 and del10 generated the novel C termini with shortened calcium-buffering sites of the C domain and absent KDEL (Fig. 1a). Correspondingly, expression of the N-terminal side of WT CALR protein was comparable in BM nuclear cells of *Calr*$^{ins2/WT}$ mice, *Calr*$^{del10/WT}$ mice, and *Calr*$^{WT/WT}$ mice, whereas expression of the C-terminal side of WT CALR protein was similarly lower in BM cells of *Calr*$^{ins2/WT}$ mice and *Calr*$^{del10/WT}$ mice compared to *Calr*$^{WT/WT}$ mice (Fig. S1a).

In human MPNs, del52 (type 1) and ins5 (type 2) account for 80% of the *CALR* mutations [11,12], and other frameshifts are classifiable as type 1- or 2-like mutations. The negatively charged AA stretches that remain after codon A352 in mutant CALR – and associated isoelectric point (pI) shifts – define the mutations: type-1 (p.L367Tfs*46) and type 1-like CALR mutations retain only the first stretch of negatively charged AAs with pI >11.5, whereas there are a few remaining negatively charged AA stretches in type-2 (p.K385Nfs*47) and type 2-like mutations, in which pI values are lower (approximately 10.0-11.0) than type 1-like mutations [2]. Previously reported knock-in mice carried murine *Calr* del19 [13], del52, or del61 [14], with only one negatively charged AA stretch representing type 1-like mutations, although pI values were lower than human type 1-like CALR mutations. In contrast, we considered *Calr* ins2 and del10 are classifiable as type 2-like mutations because of 2 remaining negatively charged AA stretches with pI values ranging within the level of type 2-like mutations in peptides of murine CALR ins2 and del10 (Fig. S1b, c). With both the ins2

and del10 frameshifts, identity and similarity of the peptides were higher, and gaps were less, with the peptide of human type-2 (ins5) frameshift, compared to the peptide of human type-1 (del52) frameshift (Fig. S1d, e).

Hematopoietic phenotypes in knock-in mice

Body weights of both *Calr*$^{ins2/WT}$ mice and *Calr*$^{del10/WT}$ mice were similar to those of *Calr*$^{WT/WT}$ mice (Fig. S2a), whereas spleens were slightly heavier in *Calr*$^{ins2/WT}$ mice compared to *Calr*$^{WT/WT}$ mice (Fig. S2b). Compared to WT mice, PB platelet and leukocyte counts were significantly increased in *Calr*$^{del10/WT}$ mice, whereas PB cell counts did not differ between *Calr*$^{ins2/WT}$ mice and *Calr*$^{WT/WT}$ mice (Fig. 1b). BM cell counts were not different between *Calr*$^{ins2/WT}$ mice or *Calr*$^{del10/WT}$ mice and *Calr*$^{WT/WT}$ mice (Fig. S2c), but both *Calr*$^{del10/WT}$ mice and *Calr*$^{ins2/WT}$ mice exhibited increased ratios of BM myeloid cells compared to *Calr*$^{WT/WT}$ mice (Fig. S2d). Histologic study showed increased megakaryocytes in *Calr*$^{del10/WT}$ mice compared to *Calr*$^{WT/WT}$ mice in BM and spleen (Fig. S2e-h). Megakaryocytes were also slightly increased in spleens of *Calr*$^{ins2/WT}$ mice (Fig. S2f, h). However, the overall structural integrity of spleens was preserved in both models. Fibrosis was not detected in BM of *Calr*$^{del10/WT}$ mice or *Calr*$^{ins2/WT}$ mice at 3 months of age and over 18 months of observation. Therefore, *Calr*$^{del10/WT}$ mice developed ET, consistent with previous knock-in or transgenic mouse models carrying frameshifted *CALR* showed ET or, rarely, myelofibrosis [15,16].

STAT3 phosphorylation and cell-surface TpoR expression in hematopoietic cells with murine *Calr* mutations

STAT3 was significantly more phosphorylated in BM nuclear cells, suspended without exogenous cytokines, of both *Calr*$^{del10/WT}$ mice and *Calr*$^{ins2/WT}$ mice compared to *Calr*$^{WT/WT}$ mice (Fig. S3a). As an upstream event of the JAK-STAT pathway, the presence of CALR protein binding to TpoR on cell surfaces is essential to activate the JAK-STAT pathway for frameshifted CALR [17–19]. Therefore, we studied expression of TpoR in lineage^{-} hematopoietic progenitor cells. The overall expression of cell-surface TpoR was similar or

lower on lineage^{-} cells of *Calr*$^{del10/WT}$ mice or *Calr*$^{ins2/WT}$ mice compared to *Calr*$^{WT/WT}$ mice, respectively (Fig. S3b). This is consistent with the previous *in vitro* finding that the presence of frameshifted murine *Calr* downregulates cell-surface TpoR expression but not the total cellular TpoR level, probably due to internalization from the cell surface or trafficking defects in cells of mutant CALR [19]. We also attempted to detect individual cells expressing both TpoR and CALR protein on surfaces of lineage^{-} progenitor cells. To evaluate CALR protein both in WT mice and KI mice, we used a fluorescent-conjugated antibody specific for intact N termini of not only WT but also mutated CALR proteins (Table S3). Cells expressing cell-surface CALR were detected almost exclusively in the TpoR^{+} fraction, and proportions of cell-surface TpoR^{+} cells were higher in lineage^{-}CALR^{+} cells of *Calr*$^{del10/WT}$ mice compared to those of *Calr*$^{WT/WT}$ mice (Fig. S3c). In *Calr*$^{del10/WT}$ mice, proportions of TpoR^{+} cells were also higher in lineage^{-}CALR^{+} cells than lineage^{-}CALR^{-} cells.

Because ET-like phenotype and pulmonary hypertension (PH) were present in *Calr*$^{del10/WT}$ mice and in del-R, we investigated TpoR expression in BM and lung, and thromboembolism and hypermetabolic state which may play pathogenic roles in MPNs [20]. TpoR^{+} cells were similarly observed in BM of *Calr*$^{del10/WT}$ mice and *Calr*$^{WT/WT}$ mice in immunofluorescent slides (Fig. S3d). By contrast, there were few TpoR^{+} cells in the lungs of both BMT recipients from *Calr*$^{del10/WT}$ mice (del-R) and those from *Calr*$^{WT/WT}$ mice (WT-R), even after the exposure to the chronic hypoxia (Fig. S3e). Lung histology showed no evidence of thromboembolism. Total cholesterol concentrations were rather decreased (P < 0.01) in the *Calr*$^{del10/WT}$ mice (37.1 ± 3.3 mg/dL, N = 3) compared to *Calr*$^{WT/WT}$ mice (58.9 ± 8.8 mg/dL, N = 5), as well as *Jak2*V617F^{+} mice with arterial sclerosis [21], suggesting that elevation of cholesterol concentration was not associated with the vascular remodeling at least in these mice.

Gene expression changes due to *CALR* mutations

To clarify the basis of MPN-like phenotypes and pulmonary hypertension (PH) of our knock-in mice, we performed RNAseq for LSK cells. Gene expressions of LSK cells were affected by ins2 and del10 frameshifts (Fig. S6a). Among differentially expressed genes, most shared

upregulation or downregulation between in *Calr*$^{del10/WT}$ mice and *Calr*$^{ins2/WT}$ mice (Fig. S6b), suggesting a similarity in the gene expression profiles of LSK cells between in these mice. In the comparison analysis of RNAseq results for canonical pathways, STAT3 and p38 MAPK signaling pathways, which have been reported to be involved in the pathogenesis of CALR-mutated MPNs [22,23], were commonly upregulated in LSK cells of *Calr*$^{ins2/WT}$ mice and *Calr*$^{del10/WT}$ mice, relative to those of *Calr*$^{WT/WT}$ mice (Fig. S6c). Interestingly, *Calr* frameshifts also activated cardiac-hypertrophy pathway (Fig. S6c), which included upregulation of *Edn-1* (*Endothelin-1*) in LSK cells of *Calr*$^{del10/WT}$ mice compared to *Calr*$^{WT/WT}$ mice (20.37-fold, Fig. S6d). GSEA of RNAseq results also showed upregulation of the JAK-STAT pathway in LSK cells of both *Calr*$^{ins2/WT}$ mice and *Calr*$^{del10/WT}$ mice (Fig. S6e).

We next studied effect of mutant human CALR expression on macrophages, because the expression of *Endothelin-1*, which is an important vasoactive peptide involving pulmonary arterial (PA) remodeling in PH [20,24], was increased in the lungs of del-R compared to WT-R under chronic hypoxia (Fig. 2a, b, Fig. S5), as well as LSK cells and LPS-stimulated macrophages from *Calr*$^{del10/WT}$ mice (Fig. 2f, g; Fig. S6d). Introduction of *CALR*-del52, as well as exposure to hypoxia, resulted in increased levels of *Endothelin-1* in a macrophage cell line (Fig. S6f, g). There was no difference between *Calr*$^{ins2/WT}$ mice and *Calr*$^{WT/WT}$ mice in levels of *Endothelin-1* expressions in LSK cells. However, these findings suggest the possibility that inflammatory stimuli in addition to the *CALR* mutation and hypoxic condition in some contexts led to *Endothelin-1* expression in macrophages that contributed to PH with PA remodeling in del-R.

Supplementary references

1. Hashimoto M, Takemoto T. Electroporation enables the efficient mRNA delivery into the mouse zygotes and facilitates CRISPR/Cas9-based genome editing. Sci Rep. 2015;5:11315.

2. Pietra D, Rumi E, Ferretti V V, Buduo CA Di, Milanesi C, Cavalloni C, et al. Differential clinical effects of different mutation subtypes in CALR-mutant myeloproliferative neoplasms. Leukemia. 2016;30:431–8.

3. Madeira F, Park YM, Lee J, Buso N, Gur T, Madhusoodanan N, et al. The EMBL-EBI search

and sequence analysis tools APIs in 2019. Nucleic Acids Res. 2019;47:W636–41.

4. Ueda K, Ikeda K, Ikezoe T, Harada-Shirado K, Ogawa K, Hashimoto Y, et al. Hmga2 collaborates with JAK2V617F in the development of myeloproliferative neoplasms. Blood Adv. 2017;1:1001–15.

5. Sugimoto K, Yokokawa T, Misaka T, Nakazato K, Ishida T, Takeishi Y. Senescence Marker Protein 30 Deficiency Exacerbates Pulmonary Hypertension in Hypoxia-Exposed Mice. Int Heart J. 2019;60:1430–4.

6. Suzuki T, Arumugam P, Sakagami T, Lachmann N, Chalk C, Sallese A, et al. Pulmonary macrophage transplantation therapy. Nature. 2014;514:450–4.

7. Shiraishi M, Shintani Y, Shintani Y, Ishida H, Saba R, Yamaguchi A, et al. Alternatively activated macrophages determine repair of the infarcted adult murine heart. J Clin Invest. 2016;126:2151–66.

8. Bolger AM, Lohse M, Usadel B. Trimmomatic: a flexible trimmer for Illumina sequence data. Bioinformatics. 2014;30:2114–20.

9. Langmead B, Salzberg SL. Fast gapped-read alignment with Bowtie 2. Nat Methods. 2012;9:357–9.

10. Robinson MD, Oshlack A. A scaling normalization method for differential expression analysis of RNA-seq data. Genome Biol. 2010;11:R25.

11. Klampfl T, Gisslinger H, Harutyunyan AS, Nivarthi H, Rumi E, Milosevic JD, et al. Somatic Mutations of Calreticulin in Myeloproliferative Neoplasms. N Engl J Med. 2013;369:2379–90.

12. Nangalia J, Massie CE, Baxter EJ, Nice FL, Gundem G, Wedge DC, et al. Somatic CALR Mutations in Myeloproliferative Neoplasms with Nonmutated JAK2. N Engl J Med. 2013;369:2391–405.

13. Shide K, Kameda T, Kamiunten A, Oji A, Ozono Y, Sekine M, et al. Mice with Calr mutations homologous to human CALR mutations only exhibit mild thrombocytosis. Blood Cancer J. 2019;9:42.

14. Balligand T, Achouri Y, Pecquet C, Gaudray G, Colau D, Hug E, et al. Knock-in of murine Calr del52 induces essential thrombocythemia with slow-rising dominance in mice and reveals key role of Calr exon 9 in cardiac development. Leukemia. 2020;34:510–21.

15. Shide K. The role of driver mutations in myeloproliferative neoplasms: insights from mouse models. Int J Hematol. 2020;111:206–16.

16. Benlabiod C, Cacemiro M da C, Nédélec A, Edmond V, Muller D, Rameau P, et al. Calreticulin del52 and ins5 knock-in mice recapitulate different myeloproliferative phenotypes

observed in patients with MPN. Nat Commun. 2020;11:4886.
17. Araki M, Yang Y, Masubuchi N, Hironaka Y, Takei H, Morishita S, et al. Activation of the thrombopoietin receptor by mutant calreticulin in CALR-mutant myeloproliferative neoplasms. Blood. 2016;127:1307–16.
18. Chachoua I, Pecquet C, El-Khoury M, Nivarthi H, Albu RI, Marty C, et al. Thrombopoietin receptor activation by myeloproliferative neoplasm associated calreticulin mutants. Blood. 2016;127:1325–35.
19. Balligand T, Achouri Y, Pecquet C, Chachoua I, Nivarthi H, Marty C, et al. Pathologic activation of thrombopoietin receptor and JAK2-STAT5 pathway by frameshift mutants of mouse calreticulin. Leukemia. 2016;30:1775–8.
20. Adir Y, Elia D, Harari S. Pulmonary hypertension in patients with chronic myeloproliferative disorders. Eur Respir Rev. 2015;24:400–10.
21. Yokokawa T, Misaka T, Kimishima Y, Wada K, Minakawa K, Sugimoto K, et al. Crucial role of hematopoietic JAK2V617F in the development of aortic aneurysms. Haematologica. 2021;In press.
22. Kollmann K, Warsch W, Gonzalez-Arias C, Nice FL, Avezov E, Milburn J, et al. A novel signalling screen demonstrates that CALR mutations activate essential MAPK signalling and facilitate megakaryocyte differentiation. Leukemia. 2017;31:934–44.
23. Araki M, Komatsu N. The role of calreticulin mutations in myeloproliferative neoplasms. Int J Hematol. Springer Japan; 2020;111:200–5.
24. Simonneau G, Gatzoulis MA, Adatia I, Celermajer D, Denton C, Ghofrani A, et al. Updated Clinical Classification of Pulmonary Hypertension. J Am Coll Cardiol. 2013;62:D34–41.
